# A circular RNA generated from an intron of the insulin gene controls insulin secretion

**DOI:** 10.1038/s41467-020-19381-w

**Published:** 2020-11-05

**Authors:** Lisa Stoll, Adriana Rodríguez-Trejo, Claudiane Guay, Flora Brozzi, Mustafa Bilal Bayazit, Sonia Gattesco, Véronique Menoud, Jonathan Sobel, Ana Claudia Marques, Morten Trillingsgaard Venø, Jonathan Lou S. Esguerra, Mohammad Barghouth, Mara Suleiman, Lorella Marselli, Jørgen Kjems, Lena Eliasson, Erik Renström, Karim Bouzakri, Michel Pinget, Piero Marchetti, Romano Regazzi

**Affiliations:** 1 Department of Fundamental Neurosciences, University of Lausanne, Lausanne, Switzerland; 2 Department of Computational Biology, University of Lausanne, Lausanne, Switzerland; 3 Interdisciplinary Nanoscience Center (iNANO) and Department of Molecular Biology and Genetics, Aarhus University, Aarhus, Denmark; 4 Islet Cell Exocytosis or Islet Pathophysiology Group, Lund University Diabetes Centre, Department of Clinical Sciences in Malmö, Lund University, Lund, Sweden; 5 Department of Clinical and Experimental Medicine, University of Pisa, Pisa, Italy; 6 UMR DIATHEC, EA 7294, Centre Européen d’Etude du Diabète, Université de Strasbourg, Fédération de Médecine Translationnelle de Strasbourg, Strasbourg, France; 7 Department of Biomedical Sciences, University of Lausanne, Lausanne, Switzerland; 8 Present Address: Department of Medicine, Weill Cornell Medicine, 413 East 69th street, New York, NY USA

**Keywords:** Long non-coding RNAs, Physiology

## Abstract

Fine-tuning of insulin release from pancreatic β-cells is essential to maintain blood glucose homeostasis. Here, we report that insulin secretion is regulated by a circular RNA containing the lariat sequence of the second intron of the insulin gene. Silencing of this intronic circular RNA in pancreatic islets leads to a decrease in the expression of key components of the secretory machinery of β-cells, resulting in impaired glucose- or KCl-induced insulin release and calcium signaling. The effect of the circular RNA is exerted at the transcriptional level and involves an interaction with the RNA-binding protein TAR DNA-binding protein 43 kDa (TDP-43). The level of this circularized intron is reduced in the islets of rodent diabetes models and of type 2 diabetic patients, possibly explaining their impaired secretory capacity. The study of this and other circular RNAs helps understanding β-cell dysfunction under diabetes conditions, and the etiology of this common metabolic disorder.

## Abstract (web summary)

Circular RNAs contribute to the regulation of β-cell specific functions. Here the authors show that a circular RNA derived from one of the introns of the insulin gene is necessary for optimal insulin secretion from pancreatic islets and that its level is reduced in the islets of diabetic subjects.

## Introduction

Circular RNAs were first observed in eukaryotes by electron microscopy over 40 years ago^1^. However, it is only recently, with the advent of modern RNA-sequencing techniques and the use of unbiased computational approaches, that circular RNAs (circRNAs) were recognized to constitute a substantial fraction of the eukaryotic transcriptome^2–4^. CircRNAs are usually generated from precursor mRNAs of protein-coding genes but lack the 5′Cap and polyA tail. Exonic and exonic-intronic circRNAs are formed by backsplicing and thus display non-colinear 3′–5′ junctions; whereas intronic circRNAs typically derive from spliced introns (lariats) circularized by non-colinear 2′–5′ junctions at the 5′ and branchpoint nucleotides. As a result, the same gene locus can produce different exon/intron-containing circRNAs. These covalently closed RNAs are stable and resistant to exonuclease degradation. They can be cell-specific and conserved between species. CircRNAs affect cellular processes by modulating gene transcription, splicing, and translation, and are emerging as important players in the development of a wide range of diseases^2–4^.

Pancreatic β-cells, located within the islets of Langerhans, secrete insulin in response to a rise in nutrients or other non-metabolic stimuli and play a central role in blood glucose homeostasis. Dysfunction or loss of these cells can lead to chronic hyperglycemia and to the development of different forms of diabetes mellitus^5^. Thus, an in depth understanding of the mechanisms controlling insulin release is essential to elucidate the etiology of this very common metabolic disease. We recently showed that human and mouse pancreatic islets express numerous circRNAs, some of which participate in the regulation of β-cell activities^6^. Indeed, the exonic circRNAs circHIPK3 and ciRS-7/CDR1as were found to modulate the expression of genes required for optimal insulin secretion and β-cell mass expansion^6,7^. Interestingly, the levels of both circHIPK3 and ciRS-7/CDR1as are diminished in the islets of diabetic *db/db* mice^6^, suggesting that lower amounts of these circRNAs contribute to the failure of β-cells to release enough insulin to cover the organisms needs in these severely obese and insulin resistant animals^8^.

In our previous study, we investigated a group of already annotated and ubiquitously expressed circRNAs^6^. However, other circRNAs may originate from important genes expressed in pancreatic islets and may have been missed in previous analyses. These circRNAs may be required for the secretory activity, proliferation, and/or survival of β-cells, and could be dysregulated in the islets of diabetic individuals and contribute to the functional β-cell mass impairment characteristic of diabetes pathophysiology^5,9^. In this work, we show an unbiased search for all potential circular transcripts present in pancreatic islets that led to the discovery of previously undetected circRNAs. Our analysis identifies a number of circRNAs originating from key β-cell genes, and reveals that a conserved intronic circRNA derived from insulin pre-mRNA is necessary for optimal insulin secretion. Indeed, its deficiency alters the expression of several genes involved in insulin exocytosis, as well as calcium signaling, and thus impairs the secretory activity of rat and human β-cells. The intronic circRNA is mainly localized in the nucleus and exerts its function by interacting with the RNA-binding protein TDP-43. Furthermore, the level of this circRNA is decreased in the islets of rodents and humans with type 2 diabetes, suggesting that it may contribute to the development of the disease.

## Results

### Identification of circRNAs generated from key β-cell genes

**Fig. 1:**
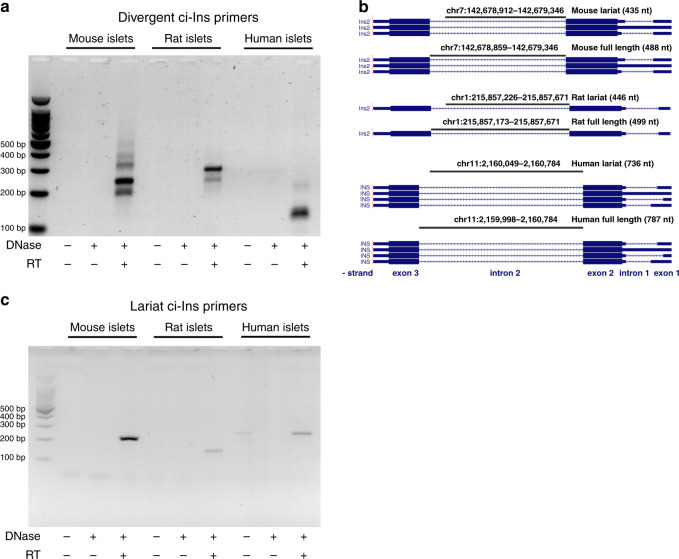
Insulin circular intronic RNAs are detectable in mouse, rat, and human islets. **a** Agarose gel electrophoresis picture showing circRNAs amplified by qPCR using divergent primers targeting the intron 2 of mouse *Ins2*, rat *Ins2*, and human *INS*. **b** Loci of sequenced qPCR products of mouse *Ins2* (mm10), rat *Ins2* (rn6), and human *INS* (hg38) intron 2 lariat and full-length circRNAs. The image was obtained using the UCSC genome browser (http://genome.ucsc.edu). **c** Agarose gel electrophoresis picture of lariat mouse and rat ci-Ins2 and human ci-INS qPCR products amplified with over-junction primers. **a**, **c** cDNA synthesis of islet RNA was performed with (+) or without (−) DNase treatment and with (+) or without (−) addition of reverse transcriptase (RT). *n* = 1 independent experiment. Source data are provided as a Source Data file.

**Fig. 2:**
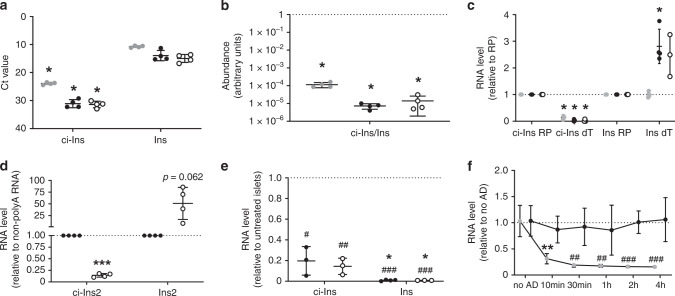
ci-Ins2 is a stable circular intronic RNA. **a**–**c** Mouse (gray circles) and rat (black circles) ci-Ins2 and *Ins2*, and human (clear circles) ci-INS and *INS* were analyzed in islet RNA samples by qPCR. The expression of the circRNAs was assessed using over-junction primers. **a** Ct values in 10 ng RNA, **b** relative circRNA/mRNA abundance (log_10_ scale), and **c** RNA levels in oligo dT (dT) cDNA relative to random primer (RP) cDNA measured by qPCR. *n* = 3 (**c**, human) and 4 (**a**–**c** (mouse and rat)) independent experiments. *p ≤ 0.001 (**a**–**c** (ci-Ins dT) and *p = 0.011 (**c**, rat *Ins2* dT) vs. *Ins2*, *INS*, or RP by two-tailed unpaired or one-sample *t*-test. **d** Rat ci-Ins2 and *Ins2* measured by qPCR in islet polyA RNA (clear circles) relative to non-polyA RNA (black circles). *n* = 4 independent experiments. ***p = 0.000025 vs. non-polyA RNA by two-tailed one-sample *t*-test. **e** Rat ci-Ins2 and *Ins2* (black circles) and human ci-INS and *INS* (clear circles) measured by qPCR in RNase R-treated islet RNA relative to untreated RNA. *n* = 3 and 4 (rat *Ins2*) independent experiments. ^#^*p* = 0.01, ^##^*p* = 0.003, and ^###^*p* < 0.0001 vs. no RNase R-treated RNA and *p = 0.04 vs. ci-Ins2 or ci-INS by two-tailed one-sample or unpaired *t*-test. **f** ci-Ins2 (black circles) and *Ins2* intron 1 (gray circles) normalized to *Hprt1*, measured by qPCR, in rat islet cells after actinomycin D treatment relative to no treatment (no AD). *n* = 5 independent experiments denoted as the mean. **p = 0.008 vs. no AD *Ins2* intron 1, ^##^*p* = 0.003 (30 min), ^##^*p* = 0.008 (1 h), ^###^*p* = 0.0003 (2 h), and ^###^*p* = 0.0001 (4 h) vs. ci-Ins2 at the corresponding time point by two-way ANOVA and Tukey post hoc test. **a**–**f** Data and mean are represented ± s.d. Source data are provided as a Source Data file.

Taking advantage of a dedicated microarray platform containing probes spanning over the predicted circular junctions of annotated transcripts, we previously identified more than 3000 circRNAs in pancreatic islets^6^. A major limitation of this methodology is that it can only detect circRNAs that are already annotated in other datasets. To circumvent this problem and obtain a comprehensive picture of all circRNAs present in islet cells, we used a two-algorithm computational approach to de novo annotate potential circular transcripts detectable in high-throughput RNA-sequencing data from mouse islets (GEO accession GSE92602)^10^. This computational approach led to the prediction of 15,925 putative circRNAs (file provided in the GEO accession GSE134699), which included circRNAs generated from key β-cell genes such as *Ins1*, *Ins2, Chga*, *Chgb*, *Gck*, *Glp1r*, *Pcsk1*, *Pcsk2*, and *Slc30a8*^5,11^ (Supplementary Table 1). Because of the essential role played by insulin in β-cell function and blood glucose homeostasis^5^, we decided to focus on circRNAs originating from the insulin genes. Since the *Ins1* gene is not conserved in humans, we elected to study in more detail the circRNAs including sequences of the insulin 2 (*Ins2*) gene, which is the rodent orthologue of the human insulin (*INS*) gene^12^. Our computational annotation identified three potential circRNAs produced from the *Ins2* gene (Supplementary Table 1). Interestingly, the predicted circRNAs included sequences belonging to intron 2. We first verified by RT-qPCR the existence of these circRNAs in mouse, rat, and human islets using divergent primers designed to amplify circularized transcripts^13,14^ (Supplementary Fig. 1). Gel electrophoresis revealed the amplification of two or more qPCR products in DNase-treated and reverse-transcribed islet RNA from each of the three species (Fig. 1a). The presence of multiple PCR products amplified with ci-Ins divergent primers may potentially be due to the recognition of multiple branchpoints as described previously^15^. Sequencing of these qPCR products indicated two common types of non-colinear junctions between species corresponding to the lariat or to the totality (full length) of the second intron of the insulin pre-mRNA (Fig. 1b and Supplementary Fig. 2). The junction loci in mouse were similar to two of the computationally predicted *Ins2* circRNAs: the lariat-derived circRNA_11718 and the full length-derived circRNA_03986 (Supplementary Table 1). We next designed qPCR over-junction primers that do not cross-react with the corresponding insulin pre-mRNA and specifically amplify the lariat-derived transcripts of the second intron of mouse or rat *Ins2* (ci-Ins2), or of human *INS* (ci-INS) (Fig. 1c). We decided to focus on the lariat-derived circRNA despite its relatively low abundance compared to its parent gene (Fig. 2a, b) since this class of circRNAs has been shown to play important regulatory roles in other cell types^13,16–18^. An estimation of the number of transcripts (Supplementary Table 2) revealed that ci-Ins2 is about five times less abundant compared to circHIPK3 (one of the most abundant circRNAs present in β-cells^6^) but is twenty times more abundant than ciRS-7 (a circRNA that has been reported to regulate insulin secretion^6,7^).

### ci-Ins2 is stable and exclusively detected in β-cells

We next verified the circularity of the ci-Ins2 and ci-INS transcripts. Since circRNAs are not polyadenylated^19^, they should not be efficiently reverse-transcribed using oligo-dT primers. Indeed, while polyadenylated linear insulin mRNA of mouse, rat, and human were equally well amplified from islet cDNAs produced with oligo-dT or with random primers, ci-Ins2 and ci-INS could be efficiently amplified only using random primers (Fig. 2c). Moreover, ci-Ins2 was poorly amplified in polyA-enriched rat islet RNA in contrast to *Ins2* (Fig. 2d). These observations confirm the absence of polyA tails in these lariat transcripts in the three species. To further verify the circularity of ci-Ins2 and ci-INS, we tested their resistance to RNase R, an exoribonuclease that digests linear but not lariat or circRNAs^20^. After treating islet RNA with this enzyme, we found that the rat ci-Ins2 is about 20 times more resistant to RNase R degradation compared to the linear *Ins2* mRNA (Fig. 2e), confirming the circularity of the intronic sequence. Similar findings were obtained with the human ci-INS and *INS* mRNA (Fig. 2e). Intron lariats are generated during splicing and are usually degraded within minutes^21^. To assess whether ci-Ins2 remains stable after *Ins2* pre-mRNA splicing, we measured its level in rat islet cells treated with the transcription inhibitor actinomycin D. We found that the level of the lariat derived from intron 2 remained constant for at least 4 h. In contrast, intron 1 of *Ins2* pre-mRNA was substantially degraded within 10 min (Fig. 2f).

**Fig. 3:**
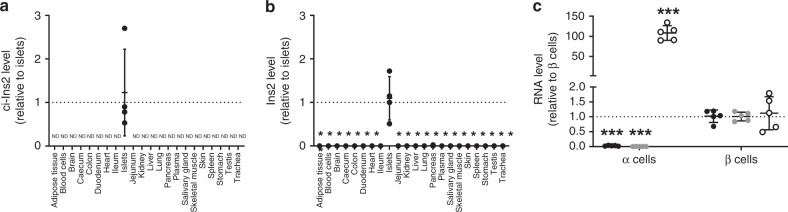
ci-Ins2 is specifically detected in β cells. **a** ci-Ins2 and (**b**) *Ins2* normalized to *Hprt1*, measured by qPCR, in rat blood cells, plasma, and 19 tissues relative to islet levels. ND = not detected. *n* = 4 independent experiments. *p < 0.0001 vs. islets by one-way ANOVA and Dunnett post hoc test. **c** ci-Ins2 (black circles), *Ins2* (gray circles), and *Gcg* (clear circles) normalized to *Hprt1*, measured by qPCR, in rat α-cells relative to β-cells. *n* = 5 independent experiments. ***p = 0.000005 (ci-Ins2), ***p = 0.0001 (*Ins2*), and ***p = 0.0002 (*Gcg*) vs. β-cells by two-tailed unpaired *t*-test. **a**–**c** Data and mean are represented ± s.d. Source data are provided as a Source Data file.

We then verified the level of ci-Ins2 and its parent gene *Ins2* in rat tissues and islet cells. As expected, *Ins2* and ci-Ins2 were only present in pancreatic islets (Fig. 3a, b). Among islet cells, ci-Ins2 was found to be highly enriched in β-cells (99% of insulin-positive cells) and was only marginally detected in the fraction containing α-cells (89% of glucagon-positive cells) (Fig. 3c). The presence of low levels of the circRNA in the α-cell-enriched fraction is most probably due to the few contaminating β-cells as small amounts of *Ins2* were also detected (Fig. 3c).

### ci-Ins2 and ci-INS are required for optimal insulin secretion

**Fig. 4:**
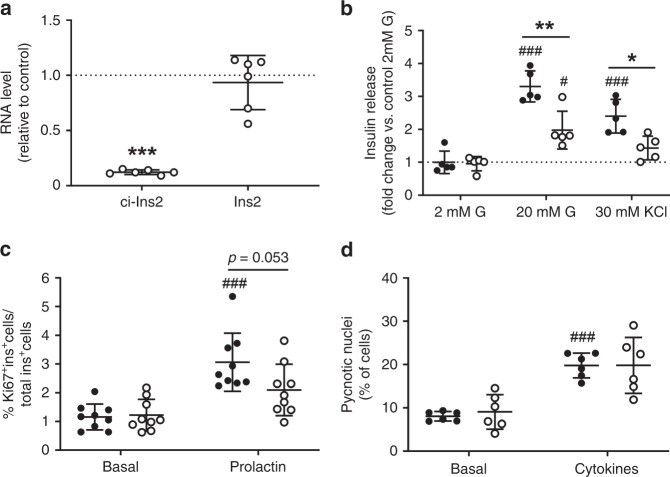
ci-Ins2 is involved in rat β-cell secretory activity. **a**–**d** Dissociated rat islet cells were transfected with control (black circles) or ci-Ins2 (clear circles) gapmeRs. Experiments were performed 48 h after transfection. **a** ci-Ins2 and *Ins2* normalized to *Hprt1*, measured by qPCR, and expressed relative to control. *n* = 6 independent experiments. ***p < 0.0001 by two-tailed one-sample *t*-test. **b** Insulin secretion in response to 2 mM glucose (G), 20 mM G, or 30 mM KCl represented as the fold change vs. control basal (2 mM G) condition. *n* = 5 independent experiments. *p = 0.018 and **p = 0.0007 as indicated, and ^#^*p* = 0.016, ^###^*p* < 0.0001 (20 mM G) and ^###^*p* = 0.0004 (30 mM KCl) vs. 2 mM G control by one-way ANOVA and Tukey post hoc test. **c** Basal and 48 h prolactin-stimulated β-cell proliferation determined by Ki67 and insulin staining. *n* = 9 independent experiments. ^###^*p* = 0.00005 vs. basal control condition by one-way ANOVA and Tukey post hoc test. **d** Basal and 24 h cytokine-induced apoptosis determined by scoring pycnotic nuclei. *n* = 6 independent experiments. ^###^*p* = 0.0002 vs. basal control condition by one-way ANOVA and Games-Howell post hoc test. **a**–**d** Data and mean are represented ± s.d. Source data are provided as a Source Data file.

**Fig. 5:**
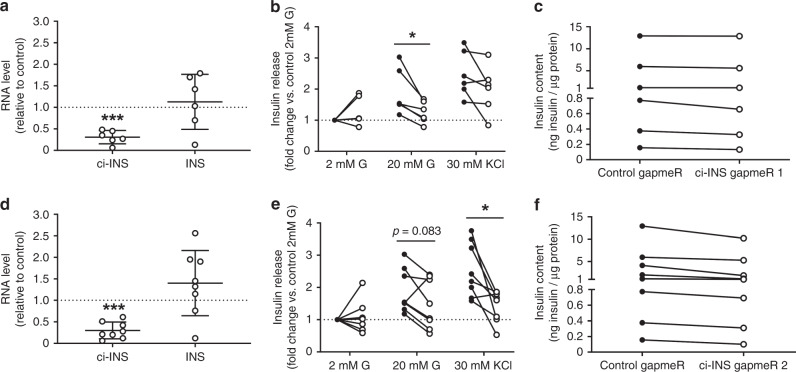
ci-INS is necessary for efficient human β-cell insulin secretion. **a**–**f** Dissociated human islet cells were transfected with control gapmeR (black circles) or with ci-INS (clear circles) gapmeR 1 (**a**–**c**) or gapmeR 2 (**d**–**f**). Experiments were performed 48 h after transfection. **a**, **d** ci-INS and *INS* normalized to *HPRT1* and *PPIA*, measured by qPCR, and expressed relative to control in (**a**) ci-INS gapmeR 1 (*n* = 6 independent experiments) or (**d**) ci-INS gapmeR 2 (*n* = 8 independent experiments). Data and mean are represented ±  s.d. ***p = 0.0001 (**a**) and ***p = 0.00002 (**d**) by a two-tailed one-sample *t*-test. **b**, **e** Insulin secretion in response to 2 mM glucose (G), 20 mM G, or 30 mM KCl represented as the fold change vs. control basal (2 mM G) condition in (**b**) ci-INS gapmeR 1 (*n* = 6 independent experiments) or (**e**) ci-INS gapmeR 2 (n = 8 independent experiments). *p = 0.018 (**b**) and *p = 0.011 (**e**) by two-tailed paired *t*-test. **c**, **f** Cellular insulin content normalized to protein content in control and (**c**) ci-INS gapmeR 1 (*n* = 6 independent experiments) or (**f**) ci-INS gapmeR 2 (*n* = 8 independent experiments). Source data are provided as a Source Data file.

We next examined the role of ci-Ins2 in the regulation of β-cell function and mass. For this purpose, we knocked down ci-Ins2 in rat islet cells using an antisense LNA oligonucleotide (gapmeR) that targets the junction of the rat circRNA and does not affect the level of the linear *Ins2* transcript (Fig. 4a and Supplementary Fig. 1). This treatment did not alter cellular insulin content (control gapmeR: 15 ± 7 vs. ci-Ins2 gapmeR: 19 ± 10 ng insulin/µg protein, *n* = 5, mean ± s.d., two-tailed unpaired *t*-test), but reduced insulin secretion in response to stimulatory glucose or KCl concentrations (Fig. 4b) and tended to reduce prolactin-stimulated β-cell proliferation (Fig. 4c). In contrast, basal insulin secretion (Fig. 4b), as well as, basal and cytokine-induced islet apoptosis (Fig. 4d) were not affected. We then assessed whether human ci-INS also regulates β-cell insulin secretion. Despite the expected variability in the secretory response of human islets obtained from different donors, silencing this circRNA with two distinct ci-INS junction gapmeRs targeting different sequences from that of rat ci-Ins2 (Fig. 5a, d and Supplementary Fig. 1) resulted in a decrease in glucose- or KCl-induced insulin secretion (Fig. 5b, e). Instead, basal insulin secretion (Fig. 5b, e) and cellular insulin content (Fig. 5c, f) were not affected by the two ci-INS gapmeRs.

**Fig. 6:**
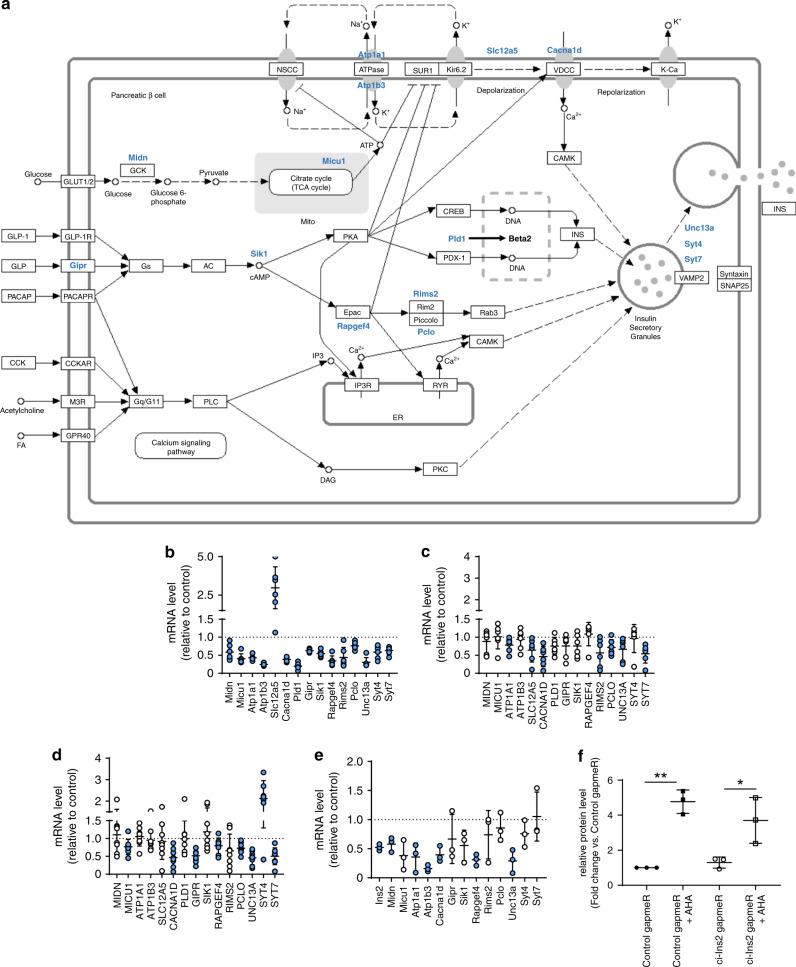
ci-Ins2 and ci-INS inhibition affect islet gene expression. **a** Downregulated and upregulated genes (blue gene names) related to insulin secretion in ci-Ins2 knockdown rat islet cells (modified KEGG pathway map 04911, https://www.genome.jp/kegg/pathway.html). Genes were selected from the differential gene expression and KEGG pathway enrichment analyses of ci-Ins2 vs. control gapmeR rat islet RNA-sequencing data (*n* = 3, *p* < 0.05, GEO accession GSE134699). **b** Expression of indicated genes normalized to *Hprt1*, measured by qPCR, in ci-Ins2 gapmeR transfected rat islet cells relative to control. *n* = 5 (*Atp1a1* and *Syt7*) and six independent experiments. **c**, **d** Expression of indicated genes normalized to *HPRT1* and *PPIA*, measured by qPCR, in (**c**) ci-INS gapmeR 1 (*n* = 6 independent experiments) or (**d**) ci-INS gapmeR 2 (*n* = 8 independent experiments) transfected human islet cells relative to control. **e** 2 h newly transcribed mRNA levels of indicated genes normalized to *Hprt1*, measured by qPCR, in ci-Ins2 gapmeR transfected rat islet cells relative to control. *n* = 3 independent experiments. **f** 2 h newly synthesized insulin protein levels, measured by ELISA, in ci-Ins2 gapmeR (clear circles and squares) transfected rat islets relative to control (black circles and squares) and treated (or not) with AHA. *n* = 3 independent experiments. **b**–**f** Data and mean are represented ± s.d. blue circles: *p* < 0.05 by two-tailed one-sample *t*-test. *p = 0.019 and **p = 0.001 by one-way ANOVA and Tukey post hoc test. Source data are provided as a Source Data file.

We next investigated the mechanisms through which ci-Ins2 regulates insulin secretion. RNA sequencing of rat islet cells transfected with the ci-Ins2 gapmeR revealed the decreased expression of 425 genes and an increase in the level of 90 genes (GEO accession GSE134699). Consistent with the observed phenotype, the downregulated mRNAs were enriched for transcripts originating from genes involved in insulin secretion. In contrast, upregulated genes were mainly related to the p53 signaling pathway (GEO accession GSE134699). Altered mRNA levels of several key genes involved in insulin secretion from glycolysis to exocytosis^22–34^ were validated by qPCR (Fig. 6a, b). These included mRNAs coding for Ca^2+^ sensors (*Syt4*, *Syt7*, and *Pclo*), Ca^2+^ channel subunits (*Cacna1d*), Na^+^/K^+^ ATPase subunits (*Atp1a1* and *Atp1b3*), and components involved in small GTPase signaling (*Rapgef4* and *Rims2*) and in the recruitment of insulin granules for exocytosis (*Unc13a*). *SYT7*, *PCLO*, *CACNA1D*, and *UNC13A* were also downregulated after knocking down ci-INS in human islets with either gapmeR 1 or 2 (Fig. 6c, d).

Intronic circRNAs have been proposed to favor the transcription of their parent genes and probably of other target genes^13^. To assess whether low ci-Ins2 levels affect the transcription of *Ins2* and of genes necessary for insulin secretion, we transfected rat islet cells with a ci-Ins2 gapmeR and two days later treated them for 2 h with 5-ethynyl uridine to tag newly transcribed RNAs. In the case of *Midn*, *Atp1a1*, *Atp1b3*, *Cacna1d*, *Rapgef4*, and *Unc13a*, total mRNAs and newly synthesized transcripts were reduced to the same extent (Fig. 6b, e), indicating that at least part of the effect of ci-Ins2 may be mediated at the transcriptional level. Although the overall *Ins2* mRNA was unchanged (Fig. 4a), we found that inhibition of ci-Ins2 reduced the quantity of newly synthesized *Ins2* mRNA (Fig. 6e). The discrepancy between these two observations may be explained by the high stability (half-life ≥29 h)^35^ and abundance of the *Ins2* mRNA (second most expressed gene in islets, GEO accession GSE134699). Therefore, we assessed whether silencing of ci-Ins2 affects the amounts of newly synthesized insulin. For this purpose, newly synthesized proteins of rat islet cells transfected with control or ci-Ins2 gapmeRs were labeled with L-Azidohomoalanine (AHA). Although we observed a tendency for a decrease in newly synthesized insulin upon ci-Ins2 silencing, the effect did not reach statistical significance (Fig. 6f). This suggests that the reduction in insulin mRNA level may be insufficient to significantly impact on insulin content.

**Fig. 7:**
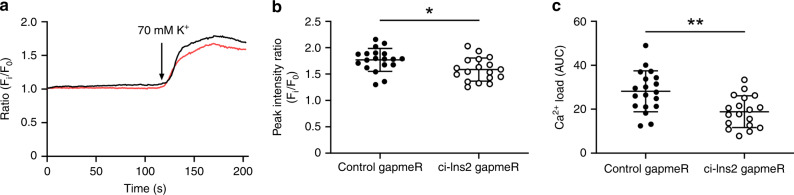
ci-Ins2 knockdown decreases depolarization-induced Ca^2+^ influx. **a**–**c** Rat islets were transfected with control (black circles) or ci-Ins2 (clear circles) gapmeRs and dissociated into single cells. Experiments were performed 48 h after transfection. **a** Representative Ca^2+^ imaging in control (black line) or ci-Ins2 (red line) gapmeRs. **b** Ca^2+^ peak intensity and **c** integrated Ca^2+^ load after K^+^ stimulation. *n* = 18 (ci-Ins2 gapmeR) and 19 (control gapmeR) cells from two independent experiments. Data and mean are represented ± s.d. *p = 0.015 and **p = 0.002 by two-tailed unpaired *t*-test. Source data are provided as a Source Data file.

Decreased expression of the voltage-dependent Ca^2+^ channel subunit *Cacna1d* or *CACNA1D* after inhibition of ci-Ins2 or ci-INS (Fig. 6b–d) suggests a defect in Ca^2+^ signaling. Consistent with this hypothesis, ci-Ins2 knockdown led to a reduction in Ca^2+^ peak and integrated load in the presence of stimulatory and membrane-depolarizing K^+^ concentrations (Fig. 7a–c).

**Fig. 8:**
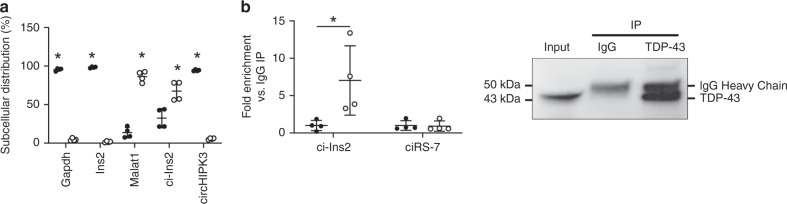
ci-Ins2 predominantly localizes to the nucleus and interacts with the RBP TDP-43. **a** Expression level of indicated genes, measured by qPCR, in nuclear (clear circles) and cytosolic fractions (black circles) of MIN6B1 cells. *n* = 4 independent experiments. **b** RNA immunoprecipitation (IP) of TDP-43 or control IgG in MIN6B1 cell lysates. *Left*: ci-Ins2 and ciRS-7 RNA levels, measured by qPCR, in immunoprecipitated samples relative to IgG. TDP-43 IP (clear circles) and IgG IP (black circles). *n* = 4 independent experiments. *Right:* Representative image of immunoprecipitated samples immunoblotted for TDP-43. Data and mean are represented ± s.d. *p ≤ 0.01 (**a**) and *p = 0.04 (**b**) by two-tailed unpaired *t*-test. Source data are provided as a Source Data file.

Next, we attempted to determine the mechanism(s) by which ci-Ins2 regulates the transcription of their target genes. To exert their cellular functions, circRNAs can bind microRNAs (miRNAs) or RNA-binding proteins (RBPs)^2–4^, and could potentially also bind transcription factors (TFs). How circRNAs act is probably dependent on their subcellular localization. In general, exonic circRNAs, such as circHIPK3, which reside in the cytoplasm have been shown to bind miRNAs, in contrast to intronic circRNAs, which localize to the nucleus, and thereby rather interact with nuclear RBPs^16,36,37^. A nuclear/cytoplasmic fractionation performed in the murine β-cell line MIN6B1 revealed that, as expected, *Gapdh, Ins2,* and circHIPK3 were localized in the cytoplasm while the lncRNA Malat1 was detected in the nucleus^38^ (Fig. 8a). Interestingly, we found that ci-Ins2 predominantly resides in the nucleus and to a lesser extent in the cytoplasmic fraction (65% vs. 35%, *p* = 0.0074, *n* = 4, Fig. 8a).

We next used a computational approach to search for potential binding sites of miRNAs, TFs, and RBPs in ci-Ins2 and ci-INS genomic sequences, and performed an enrichment analysis of miRNAs and TFs targeting the qPCR-validated genes differentially expressed after ci-Ins2 or ci-INS inhibition (Supplementary Tables 3–7, Fig. 6b–d). Since it is present in the cytoplasm, ci-Ins2 could potentially exert its action by sponging miRNAs. However, among the miRNAs predicted to target the genes deregulated upon ci-Ins2 silencing, only one potential binding site for miR-139-3p, miR-188-5p, miR-296-5p, and miR-744 was identified in ci-Ins2 or ci-INS sequences (Supplementary Tables 3, 4). These miRNAs are barely detected in rodent and human islets (our unpublished data) and there is no experimental evidence indicating that these miRNAs may be involved in the regulation of insulin secretion.

Although the interaction between intronic circRNAs and TFs remains to be established, we also searched for possible TF binding sites. We found that ci-Ins2 or ci-INS contain sequences predicted to bind GLI2, SMAD3, and SMAD4 (Supplementary Tables 5, 6). These TFs have been shown to modulate various genes involved in insulin secretion^39–41^.

Computational analysis revealed that ci-Ins2 can potentially interact with several RBPs (Supplementary Table 7). In particular, we found that mouse ci-Ins2 is predicted to bind to the TAR DNA-binding protein 43 kDa (TDP-43, encoded by *Tardbp*). If one or few binding site mismatches are allowed the interaction between TDP-43 and mouse ci-Ins2 appears to also be conserved for rat ci-Ins2 and human ci-INS. Among the potential binding partners, TDP-43 stand out as the best candidate to mediate the actions of ci-Ins2. In fact, the knockout of this RBP in β-cells has recently been shown to result in defective insulin secretion^42^. Second, silencing of TDP-43 and ci-Ins2 results in a decrease in the expression of a common set of genes and in particular of genes involved in Ca^2+^ signaling and insulin exocytosis^42^. Third, TDP-43 has already been shown to interact with intron lariats^16^. None of the other RBPs identified in the computational analysis possesses these properties. We nevertheless do not exlude that other RBPs may mediate part of the effect of ci-Ins2.

To assess whether the blunted insulin secretion observed in our model is mediated by the interaction with TDP-43, we compared expression changes upon ci-Ins2 down-regulation in rat islets (GEO accession GSE134699) with those in MIN6 cells depleted from TDP-43 (GEO accession GSE125424). We identified 18 orthologous mouse-rat pairs whose expression change in the same direction in both models, including *Cacna1c, Pclo Pld1*, and *Rapgef4* (Supplementary Table 8). Of note, genes consistently differentially expressed between experiments are enriched in calcium ion regulated exocytosis (*p* = 0.015) and insulin secretion (*p* < 0.03) (Supplementary Table 9). To experimentally validate the predicted interaction with TDP-43, we immunoprecipitated the RNAs associated with this RBP from MIN6B1 cell lysates and found a significant enrichment of ci-Ins2. This interaction was specific since ciRS-7, an exonic circRNA involved in the control of insulin secretion, was not pulled-down by the TDP-43 antibody (Fig. 8b). Taken together, these results suggest that at least part of the effect of ci-Ins2 on the expression of genes involved in calcium signaling and insulin release is mediated through the interaction with TDP-43.

### ci-Ins2 and ci-INS levels are reduced in type 2 diabetes

**Fig. 9:**
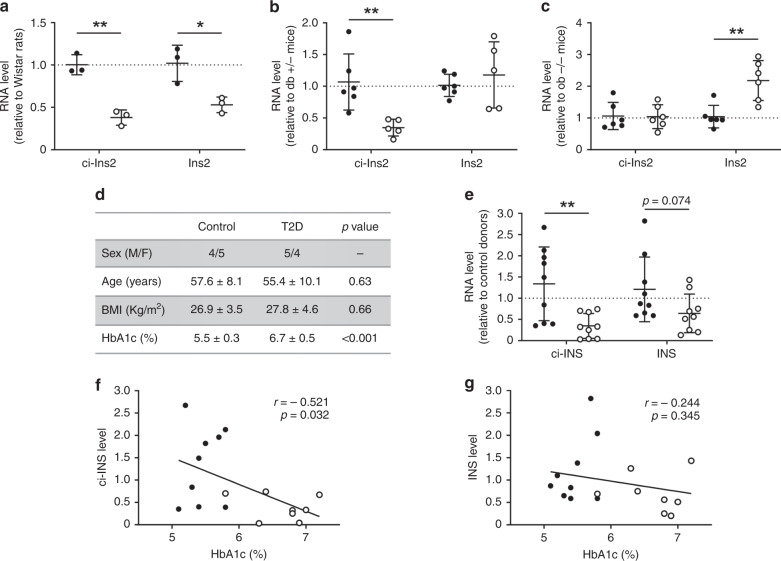
ci-Ins2 and ci-INS levels are low in type 2 diabetes rodent models and human donors. **a**–**c** Islet ci-Ins2 and *Ins2* normalized to *Hprt1*, measured by qPCR, and expressed relative to control in (**a**) type 2 diabetes Goto-Kakizaki (clear circles) relative to Wistar (black circles) rats (*n* = 3), **b** type 2 diabetes *db* +/+ (clear circles) relative to *db* +/− (black circles) mice (*n* = 5 (*db* +/+) and 6 (d*b* +/−), and (**c**) *ob* +/+ (clear circles) relative to *ob* −/− (black circles) mice (*n* = 6). **d** Characteristics of human donors (*n* = 8 (type 2 diabetes (T2D) HbA1c) and 9, Supplementary Table 10). **e** Islet ci-INS and *INS* normalized to *HPRT1* and 18S, measured by qPCR, in type 2 diabetes (clear circles) relative to control (black circles) donors (*n* = 9). **a**–**e** Data and mean are represented ± s.d. *p = 0.022 and **p ≤ 0.01 by two-tailed unpaired *t*-test. **f**, **g** Correlation of (**f**) ci-INS and (**g**) *INS* relative islet RNA levels with HbA1c in control (black circles) and type 2 diabetes (clear circles) donors (*n* = 8 (type 2 diabetes) and 9 (control)). *p* < 0.05 by two-tailed Pearson test was considered significant. Source data are provided as a Source Data file.

We then assessed whether the level of ci-Ins2 and its parent gene *Ins2* are modified in the islets of type 2 diabetes models exhibiting impaired insulin secretion. The level of ci-Ins2 and *Ins2* was indeed reduced in the islets of diabetic Goto-Kakizaki (GK) rats, a model in which type 2 diabetes develops in the absence of obesity^43^ (Fig. 9a). ci-Ins2 was also reduced in obese and hyperglycemic *db/db* mice but remained unchanged in obese but normoglycemic *ob/ob* mice^8^, while *Ins2* was unchanged in *db/db* mice and even increased in *ob/ob* mice (Fig. 9b, c). These results demonstrate that the changes in ci-Ins2 do not simply reflect those of its parent gene. Similar measurements were performed in human islets from type 2 diabetes donors displaying high HbA1c levels and compared to age- and BMI-matched non-diabetic controls (Fig. 9d and Supplementary Table 10). ci-INS levels were significantly lower and *INS* tended to be decreased in the islets obtained from type 2 diabetic patients relative to controls (Fig. 9e). Furthermore, ci-INS levels were inversely correlated with HbA1c (Fig. 9f), whereas those of *INS* were not (Fig. 9g). These findings suggest that inappropriate levels ci-INS, or its rodent ortholog ci-Ins2, may contribute to β-cell dysfunction in type 2 diabetes^8,43,44^.

## Discussion

Insulin secretion plays a central role in the control of blood glucose homeostasis and defects in this process promote the appearance of diabetes mellitus. Thus, a better understanding of the molecular mechanisms governing the release of this essential hormone is of paramount importance to elucidate the causes of this very common metabolic disorder. Recent findings from different laboratories, including ours, have shown that non-coding RNAs contribute to the fine-tuning of insulin release^6,7,45,46^. Here, we show that the insulin transcript itself is a source of circRNAs that insure the release of appropriate amounts of the hormone.

An increasing number of studies has reported the presence and the functional involvement of circRNAs in a wide variety of cells^2–4^, including pancreatic β-cells^6,7,47^. In this study, we searched for other relevant circRNAs involved in the control of β-cell functions using a computational approach permitting de novo annotation of all potential circRNAs detectable in our mouse islet high-throughput RNA-sequencing data^10^. This annotation predicted the presence of a large number of circRNAs derived from the sequences of key genes encoding proteins involved in insulin biosynthesis and secretion^5,11^. These circRNAs were not detected in our previous study that used a microarray approach and included mostly ubiquitously expressed transcripts^6^. It should be pointed out that in this study the samples used for RNAseq analysis were not pre-treated with RNase R, which may potentially introduce a bias. However, this may also be the case when RNase R treatment is performed^48^. Indeed, while incubation of total RNA with RNase R degrades linear transcripts and favors the detection of circRNAs, this treatment may decrease the chances to detect lowly abundant circRNAs and overestimate the level of intron-derived circRNAs due to lariats being resistant to the treatment. Thus, to obtain a comprehensive view of all potential circRNAs expressed in islets, we elected to perform data-analysis on untreated samples. The circularity and the relative abundance of circRNAs were then confirmed by RNase R treatment and qPCR. Recently, the presence of exonic circRNAs produced from *CHGA*, *PCSK2*, and *SLC30A8* genes was also predicted by the annotation of RNA-sequencing data from human α- and β-cells by Kaur et al.^47^. However, intronic circRNAs were not investigated in the Kaur study. Despite a few differences, probably linked to the higher sequencing depth of our data and to the use of different species, these findings confirm that circRNAs can be generated from key islet genes.

Here, we elected to focus on intronic circRNAs derived from the *Ins2* transcript and to study in more detail the circRNA formed by the intron 2 lariat that is detectable in both rodent (ci-Ins2) and human islets (ci-INS). CircRNAs originating from the *Ins1* gene were also detected in our computational analysis but were not investigated in this study. The *Ins1* gene is expressed in rodents and is not conserved in humans^49^. Moreover, in contrast to the circRNAs originating from *Ins2*, the transcripts originating from the *Ins1* gene are exonic. Thus, if they have any role in β-cells, they would likely have a different mode of action. As expected, this circular transcript generated from the processing of the insulin pre-mRNA was only detected in pancreatic β-cells. Lariats are formed during pre-mRNA splicing but are generally linearized by the RNA-debranching enzyme DBR1 and subsequently degraded by RNA nucleases^21,50,51^. Indeed, inactivation or depletion of DBR1 results in global lariat accumulation. Interestingly, some lariats can escape debranching, as well as degradation, and become stable functional circRNAs^13^. Accordingly, rat ci-Ins2 was resistant to RNase R degradation, as was human ci-INS, and was long lived. However, the mechanisms permitting these circularized transcripts to escape debranching and degradation remain to be defined.

Our data indicate that rat ci-Ins2 is not essential for β-cell survival and has only minor, if any, effects on proliferation. Instead, deficiency of this lariat in rat and human islet cells results in blunted insulin release in response to nutrients (glucose) and/or membrane-depolarizing compounds (KCl). Impaired insulin secretion can be explained by changes in the expression of key genes involved in the secretory pathway of β-cells. Indeed, silencing of rat ci-Ins2, or human ci-INS, was associated with a reduction in a striking number of mRNAs encoding essential components of the secretory machinery of β-cells. These include Ca^2+^ channel subunits regulating the entry of the cation upon membrane depolarization, Ca^2+^ sensors, and different targets and regulators of Rab3 GTPases. Most of these changes were consistent in both species and were reproduced with different gapmeR sequences, confirming the specificity of the effect. Moreover, Ca^2+^ influx stimulated by K^+^ depolarization was decreased in ci-Ins2 knockdown islets in agreement with the downregulated expression of voltage-dependent Ca^2+^ channels.

Little is known about the mode of action of intronic circRNAs. Lariat-derived circRNAs have been shown to act as decoys of RBP^16^, by regulating pri-miRNA processing to mature miRNAs^17^, or as regulators of RNA splicing^18^. Moreover, intronic circRNAs were reported to be preferentially localized in the nucleus and to directly interact with the transcriptional machinery^13^. Accordingly, ci-Ins2 was found to be more abundant in the nucleus and part of its effects appear to be exerted at the transcriptional level by interacting with the RBP TDP-43. Nuclear depletion of this RBP is the histopathological hallmark of amyotrophic lateral sclerosis (ALS). Since ALS patients display impaired glucose homeostasis, Araki and colleagues investigated the potential involvement of TDP-43 in the regulation of insulin secretion. They observed that ALS subjects have reduced early-phase insulin release, which correlates with a decrease in TDP-43 levels in β-cells. Moreover, knock-down of the *Tardbp* gene (encoding TDP-43) in mice did not affect insulin content but resulted in blunted insulin secretion in vivo and ex vivo and in a reduced expression of *Cacna1c*. Finally, overexpression of Cav1.2 restored early-phase insulin secretion in *Tardbp-*depleted MIN6 cells. Among the genes deregulated in islet cells upon ci-Ins2 silencing, 18 are common to *Tardbp*-depleted MIN6 cells and are related to calcium ion regulated exocytosis and insulin secretion pathways. While upon silencing of ci-Ins2 and TDP-43 most genes directly involved in the regulation of insulin secretion display changes in the same direction (in particular *Rapgef4*, *Pld*1, *Pclo,* and *Cacna1c*), some of the other genes do not. It should be pointed out that in our study, we measured gene expression in rat islet cells while Araki et al.^42^ analyzed the impact of TDP-43 silencing in the mouse insulin-secreting cell line MIN6. Moreover, our analysis was carried out by RNA sequencing while Araki et al. used a microarray approach. These experimental differences may potentially explain part of the discrepancies. Moreover, TDP-43 is known to regulate the expression of a large set of genes^52^ and most of the effects of this RBP occur also in cells that do not express insulin. Thus, even in β-cells part of these changes are certainly independent of ci-Ins2. Taken together, our findings suggest that at least part of the effect of ci-Ins2 may be mediated by TDP-43. The precise mechanisms through which ci-Ins2 and TDP-43 regulate insulin release remains to be elucidated. In principle, ci-Ins2 could act both as a co-activator or a decoy for TDP-43. Since TDP-43 is known to associate with a large number of transcripts, ci-Ins2 could potentially guide TDP-43 towards the interaction with certain transcripts but prevent its binding to others. However, our data would fit more easily with a model in which ci-Ins2 functions as a co-activator because the expression of most genes involved in insulin secretion is reduced upon silencing of TDP-43 or ci-Ins2.

Our computational predictions suggest that ci-Ins2/ci-INS contains potential binding sites for the TFs SMAD4, SMAD3, and GLI2. Based on our analysis it is not possible to determine whether these TFs would bind the circRNA or the corresponding genomic sequence. However, since some TFs have been proposed to bind to RNA^53,54^ we included the computational analysis to provide the reader with a comprehensive picture of all possible partners of the circRNA. SMAD3 and GLI2 were reported to impair insulin biosynthesis and glucose-stimulated secretion, partly through repressing insulin transcription^39,40^. In the present study, silencing of ci-Ins2 resulted in a small but significant decrease in newly transcribed *Ins2*, but did not affect insulin content nor insulin biosynthesis. Since it has been reported that pre-existing insulin mRNA is preferentially translated upon glucose stimulation^55^ we cannot fully exclude that, as previously shown for other intronic circRNAs^13^, prolonged reduction in ci-Ins2 affects the expression of its parent gene and would result, over a long period, in a decrease in β-cell insulin content.

A fraction of ci-Ins2 appears to reside in the cytoplasm, fitting with the observations that lariats can in some circumstances be exported to this cellular compartment. Thus, we cannot exclude that ci-Ins2 could also be involved in the regulation of some post-transcriptional events as it has been demonstrated for exonic circRNAs. However, the effect of ci-Ins2 on insulin secretion is unlikely to be mediated by sponging the miRNAs that are predicted to interact with this intronic circRNA, since they are barely expressed in β-cells and none of them has been reported to play important roles in insulin secretion.

Interestingly, the level of ci-Ins2 is not modified in normoglycemic obese mice, but is reduced in the islets of rodent models of type 2 diabetes as observed for circHIPK3 and ciRS-7^6^. This finding was confirmed in human. Indeed, the level of ci-INS was found to be significantly lower in the islets of type 2 diabetes donors and to be inversely correlated to HbA1c levels. This suggests that reduced ci-Ins2 and ci-INS may contribute to β-cell failure in rodent and human type 2 diabetes^8,43,44^.

**Fig. 10:**
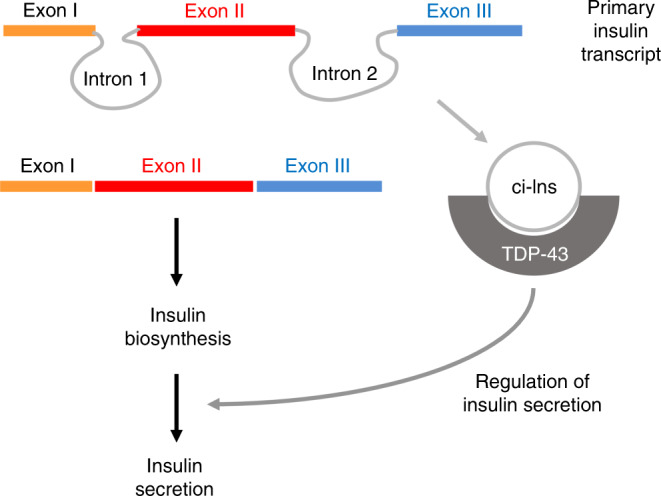
ci-Ins is required for optimal insulin secretion. In β-cells, the primary insulin transcript is processed to generate the mature mRNA that is translated for insulin biosynthesis. In parallel, the second intron excised from the primary insulin transcript forms a stable circular RNA (ci-Ins2/ci-INS) that associates with the RBP TDP-43 and controls the expression of genes necessary for optimal insulin release in response to secretory stimuli.

In conclusion, our data indicate that the primary transcript of the insulin gene is not only providing the code for insulin biosynthesis, but is also generating a circRNA that is necessary to ensure an efficient release of this hormone under stimulatory conditions (Fig. 10). Future experiments will have to determine whether this phenomenon is restricted to β-cells or whether circRNAs with analogous regulatory functions are operating also in other endocrine cells. Elucidating the role of this and other circRNAs will contribute to a better understanding of the etiology of diabetes mellitus and potentially of other endocrine diseases.

## Methods

### CircRNA annotation

circRNAs were annotated in high-throughput RNA-sequencing data from mouse islets (GEO accession GSE92602)^10^ by computational detection using two separate circRNA algorithms: CIRI2^56^ and find_circ^57^. Only circRNAs detected by both algorithms were considered and two or more backsplice spanning reads were required to call circRNAs in individual samples.

### Animals

Male Wistar rats and C57Bl/6N mice aged 12 weeks were acquired from Janvier Labs, male C57BL/KsJ *db/db*, C57BL/6J *ob/ob*, and their respective control *db* +/− and *ob* −/− mice aged 13–16 weeks from the Garvan Institute breeding colonies, and female Goto-Kakizaki (GK/MolTac) and control Wistar rats aged 8 weeks from Taconic Europe. Animals were housed under standard temperature (20–24 °C) and humidity (40–70%) conditions on a 12-h light/dark cycle with free access to chow diet and water. Procedures were performed in agreement with the NIH guidelines and approved by the Swiss research council and veterinary offices, the national health and medical research council of Australia, and the ethics committee of Lund University.

### Human pancreatic islets

Human islets from male and female type 2 diabetes and aged- and BMI-matched non-diabetic control cadaver donors (Supplementary Table 10) were provided by the Human Tissue Lab of EXODIAB/Lund University Diabetes Centre through the Nordic Network for Islet Transplantation of Uppsala University. For in vitro experiments, human islets from male and female non-diabetic cadaver donors (Supplementary Table 10) were provided by the Centre Européen d’Etude du Diabète of Strasbourg University (isolation protocol authorization for scientific research #PFS12-0013), the Department of Clinical and Experimental Medicine of Pisa University, and the European Consortium for Islet Transplantation of Hôpitaux Universitaires de Genève through the JDRF award 31-2008-416 (ECIT Islet for Basic Research program). Informed consent was previously provided by all donors. Procedures complied with relevant ethical regulations and were approved by the ethics committees of the corresponding Universities.

### Pancreatic islet isolation, dissociation, and sorting

Mouse and rat islets were isolated by collagenase (Roche) digestion followed by Histopaque (Sigma) density gradient and handpicking^58^. When needed, rodent and human islets were dissociated into single cells by incubation in Ca^2+^/Mg^2+^ free phosphate-buffered saline, 3 mM EGTA, and 0.002% trypsin (Gibco) for 5–10 min at 37 °C. Enriched fractions of α- and β-cells were obtained by Fluorescence-Activated Cell Sorting (FACS) of dissociated islet cells based on β-cell autofluorescence as previously described^59,60^. FACS purity was assessed by double immunofluorescence staining and scoring of α- (glucagon-positive) and β- (insulin-positive) sorted cells using mouse anti-glucagon (Abcam ab10988, 1:1000), guinea pig anti-insulin (Dako A0564, 1:100), goat anti-mouse IgG Alexa Fluor 555 (Thermo Fisher A-21422, 1:500), and goat anti-guinea pig IgG Alexa Fluor 488 (Thermo Fisher A-11073, 1:500) antibodies. On average, β-cell fractions contained 99.1 ± 0.9% insulin-positive cells and 0.6 ± 0.6% glucagon-positive cells, while α-cell-enriched fractions contained 10.6 ± 8.2% insulin-positive cells and 88.8 ± 8.2% glucagon-positive cells. Rodent islet cells were cultured in RPMI 1640 GlutaMAX medium (Gibco) supplemented with 10% fetal bovine serum (Gibco), 100 U/mL penicillin and 100 µg/mL streptomycin (Gibco), 1 mM sodium pyruvate (Sigma), and 10 mM Hepes (Sigma). Instead, human islet cells were cultured in CMRL 1066 medium (Gibco) supplemented with 10% fetal bovine serum, 100 U/mL penicillin, and 100 µg/mL streptomycin, 2 mM L-glutamine (Gibco), and 10 mM Hepes.

### Culture of MIN6B1 cells

The murine insulin-secreting cell line MIN6B1^61^ was cultured in DMEM-Glutamax medium (Invitrogen), supplemented with 15% fetal calf serum (Gibco), 50 U/ml penicillin and 50 µg/ml streptomycin (Gibco), and 70 µM β-mercaptoethanol (Sigma). MIN6B1 cells were tested negative for mycoplasma contamination.

### Cell transfection

Dissociated rat and human islet cells were transfected using Lipofectamine RNAiMAX (Thermo Fisher) with either control or species-specific ci-Ins2 and ci-INS antisense LNA oligonucleotides (gapmeRs, Qiagen) with the following sequences: control gapmeR: 5′-AACACGTCTATACGC-3′, rat ci-Ins2 gapmeR: 5′-GCTTACAGACACATAG-3′, human ci-INS gapmeR 1: 5′-GCTCACGGACACAGGA-3′, and human ci-INS gapmeR 2: 5′-CGGTTGGCTCACGGAC-3′. Cells were then cultured for 48 h before RNA extraction or functional assays.

### Blood and tissue sampling

Rat blood was collected by post-mortem cardiac puncture and centrifuged to separate blood cells and plasma whilst tissues were dissected and immediately frozen in liquid nitrogen. Thereafter, samples were disrupted and homogenized in QIAzol lysis reagent (Qiagen) and conserved at −80 °C until RNA extraction.

### Total RNA and qPCR

Total RNA from islet cells, blood cells, plasma, and tissue samples were extracted with the miRNeasy kit (Qiagen), treated with DNase (Promega or Qiagen), and reverse transcribed with an M-MLV reverse transcriptase and random primers (Promega) or with the High Capacity cDNA kit with RNase inhibitor (Thermo Fisher). Quantitative PCR (qPCR) was performed using the SsoAdvanced Universal SYBR Green Supermix (BioRad) or the PowerUP SYBR Green Master Mix (Applied Biosystems). The number of circRNA and mRNA molecules per ng of rodent islet RNA was evaluated employing a corresponding standard curve. Primer sequences are listed in Supplementary Table 11. Selected qPCR products were loaded in agarose gels for electrophoresis, purified with the QIAquick gel extraction kit (Qiagen), and sequenced. The sequencing files were analyzed using Chromas (2.6.5).

### RNA circularity

Total RNA from islets was treated with DNase and reverse transcribed with random or oligo dT primers (Promega) followed by qPCR amplification, or incubated with or without 2.5 U/µg RNase R (Epicentre) at 37 °C for 15 min or separated into polyA RNA and non-polyA RNA (unbound to oligo dT beads) using the Poly(A)Purist MAG kit (Thermo Fisher AM1922) followed by RNA extraction, DNase treatment, reverse transcription with random primers, and qPCR amplification.

### RNA stability

Dissociated islet cells were cultured with 10 µg/mL actinomycin D (Sigma) for 10 min, 30 min, 1 h, 2 h, and 4 h, and harvested for total RNA extraction followed by DNase treatment, reverse transcription using random primers, and qPCR amplification.

### Nascent RNA pull down

Transfected islet cells were treated with 200 µM 5-ethynyl uridine (EU) for 2 h to tag newly transcribed RNA. Thereafter, cells were harvested for total RNA extraction. EU-RNA was then biotinylated and pulled down using the Click-iT Nascent RNA Capture kit (Thermo Fisher C10365). The EU-RNA captured on streptavidin beads was reverse transcribed with random primers and target RNA levels were measured by qPCR.

### Nascent insulin protein assay

After 48 h of transfection with control or ci-Ins2 gapmeRs, rat islet cells were treated for 2 h with 100 μM of L-Azidohomoalanine (AHA) (Jena Bioscience, CLK-AA005) in methionine-free RPMI medium (Thermo Fisher, A1451701) to tag newly synthesized proteins. Then, proteins were collected in 1% SDS−50 mM Tris-HCl buffer and biotinylated by click reaction using the Click-iT Protein Analysis Detection kit (Thermo Fisher, 3337). Biotinylated proteins were pulled down with an excess of streptavidin-coated beads (Dynabeads MyOne Streptavidin C1, Thermo Fisher, 65001) and eluted from the beads by boiling the samples for 3 min in 0.1% SDS in PBS. Pulled-down biotinylated insulin was quantified by ELISA (Mercodia, 10-1250-10).

### Insulin secretion and content

Transfected islet cells were first incubated at 37 °C for 45 min in a Krebs-Ringer bicarbonate buffer (KRBH) containing 25 mM HEPES, pH 7.4, 0.1% BSA, and 2 mM glucose (Sigma). Thereafter, the cells were incubated at 37 °C for 45 min in KRBH-BSA solutions with 2 mM (basal) or 20 mM (stimulatory) glucose, or with 2 mM glucose and 30 mM (stimulatory) KCl (Sigma). After incubation, supernatants were collected. The cells kept at basal glucose were harvested using acid ethanol (75% ethanol, 0.55% HCl), and those incubated at stimulatory glucose were lysed using Triton X-100 lysis buffer to determine insulin and protein contents, respectively. Insulin levels were measured by ELISA (Mercodia) and cellular protein contents by Bradford assay (Thermo Fisher).

### Ca2+ imaging

Transfected islet cells were washed with KRBH containing 2.8 mM glucose and incubated with 1 μM Fura-2 AM (Thermo Fisher) in KRBH at 37 °C for 30 min to measure intracellular Ca^2+^. The cells were then washed with KRBH and perfused with KRBH containing 70 mM K^+^ at a flow rate of 1 mL/min at 32 °C. Fura-2 AM fluorescence was monitored at 340 and 380 nm (excitation wavelengths) upon Ca^2+^ binding, and the emitted light signals were read at 510 nm. Polychrome V high speed switching monochromator on a Nikon microscope equipped with an Andor ER-BOB-100 trigger box, an Andor camera Ixon3, and the Andor iQ (2.5.1) software were used for imaging. The area under the curve (AUC) was calculated as AUC = Σ [0.5(Ratio340/380n + Ratio340/380*n*−1) × (t*n*−t*n*−1)] (1), in which tn represents the time intervals.

### β-cell proliferation

Proliferative β-cells were detected by immunostaining of transfected islet cells with rabbit anti-Ki67 (Abcam ab15580, 1:1500), guinea pig anti-insulin (Dako A0564, 1:100), goat anti-rabbit IgG Alexa Fluor 488 (Thermo Fisher A-11008, 1:500), and goat anti-guinea pig IgG Alexa Fluor 555 (Thermo Fisher A-21435, 1:500) antibodies. Coverslips were mounted on microscope glass slides with Fluor-Save mounting medium (VWR International SA) and were visualized with a Zeiss Axiovision fluorescence microscope. Prolactin (500 ng/mL, Sigma) was used to stimulate proliferation for 48 h. A minimum of 800 cells were counted per condition.

### Islet cell apoptosis

Cell apoptosis was assessed by incubating transfected islet cells with 1 mg/mL Hoechst 33342 (Thermo Fisher) at 37 °C for 3 min and scoring the fraction of cells displaying pycnotic nuclei under a fluorescence microscope. A mix of the pro-inflammatory cytokines (PeproTech) IL-1β (0.1 ng/mL), TNF-α (10 ng/mL), and IFN-γ (30 ng/mL) was used to induce cell death for 24 h. A minimum of 300 cells were counted per condition.

### RNA sequencing

Libraries from total RNA of transfected Wistar islet cells were prepared and sequenced by the Lausanne Genomic Technologies Facility with the TruSeq Stranded mRNA Library Prep kit and a HiSeq 2500 instrument for a single end run of 125 cycles using the SBS chemistry v4 (Illumina). To determine transcript expression levels, reads were mapped to the rat transcriptome (ENSEMBL 92, rn6) using STAR^62^ (2.5.3a) and quantified with RSEM^63^ (1.3.0). To identify differentially expressed genes, reads were mapped to the rat genome (rn6) using HISAT^64^ (2.1.0) and gene counts for rat genes (ENSEMBL 92, rn6) estimated using HTseq^65^ (0.10.0). Normalization and differential gene expression (FDR < 0.05) were performed using DESeq2^66^ (v.1.12.4). Gene enrichment analysis for significantly up or down-regulated genes relative to all islet expressed genes was carried out using the DAVID webserver^67^ (6.8).

The overlap and direction of changes between genes differentially expressed upon ci-Ins2 knockdown (GEO accession GSE134699) were compared to those of TDP-43 knockdown in MIN6 cells (GEO accession GSE125424^42^) (Supplementary Table 8). The Biological Process GO term for the genes that change consistency between the two experiments (Supplementary Table 9) were analyzed using the DAVID webserver^68,69^ (6.8).

### Subcellular fractionation

Nuclear and cytosolic fractions of MIN6B1 cells were obtained using the PARIS Kit (Thermo Fisher) followed by RNA extraction, reverse transcription and qPCR as previously described.

### Binding site prediction and enrichment analysis

The lariat 5′-3′ genomic sequences of mouse and rat ci-Ins2 and of human ci-INS (Source Data Fig. 1b) were used to predict putative binding sites of miRNAs, RBPs, and transcription factors (TFs) in ci-Ins2 and ci-INS. For miRNAs expressed in β-cells^6^, a custom python script based on a sliding window was utilized to detect a perfect match of miRNA seeds (6 nt) and their position relative to the 5′-end of ci-Ins2 or ci-INS sequences. For RBPs and TFs, the motif scanning tool FIMO^70^ (5.1.0) was used together with the ATtRACT^71^ and Jaspar^72^ motif databases, and the relative position, *p* value (<10^−4^) and sequence of each match are reported. To identify miRNAs and TFs targeting the differentially expressed genes shown in Fig. 6, a gene-set enrichment analysis for binding motifs was performed using Enrichr webserver^73^ (2016).

### RNA immunoprecipitation and Western Blot

RNA immunoprecipitation was performed using the Imprint RNA Immunoprecipitation Kit (Sigma). In brief, MIN6B1 cell lysates (1 × 10^7^ cells) were immunoprecipitated with 5 µg of rabbit IgG or rabbit anti-TDP43 (Abcam ab190963) antibodies, which were pre-bound to protein A agarose beads. Following an overnight incubation with respective antibodies, equal proportions of the sample volume were used for protein and RNA isolations, respectively. For Western Blot analysis, the immunoprecipitated proteins were eluted by heating the samples to 95 °C for 5 min in 2x Laemmli Buffer supplemented with β-mercaptoethanol. The samples, including a MIN6B1 input cell lysate, were run on a SDS-PAGE gel (Bio-Rad, 4561086) and transferred onto a nitrocellulose membrane (Bio-Rad, 1620117) which was subsequently blocked for 1 h in 5% BSA in TBS-T and incubated overnight at 4 °C with the anti-TDP43 antibody (1:1000 in blocking solution). Afterwards, the membrane was incubated for 1 h with the HRP-conjugated goat anti-rabbit IgG (H + L, Jackson ImmunoResearch 211-035-109, 1:10000 in blocking solution) and subsequently revealed using the ECL substrate (Pierce, 32106). For qPCR analysis, RNA molecules bound to immunoprecipitated proteins were eluted in Qiazol followed by RNA isolation, reverse transcription and qPCR as previously described.

### Statistical analysis

Data are represented as the mean or as individual values and mean ± standard deviation (s.d.). GraphPad Prism 7 and IBM SPSS Statistics 25 software were used to plot and analyze data with suitable statistical tests. Data normality was determined with a Shapiro-Wilk test, and homogeneity of variances with a Levene test. A two-tailed one-sample *t-*test was used to compare a data set to a control value settled to 1, and two-tailed paired or unpaired *t*-tests for comparing two data sets. One- or two-way ANOVA followed by Tukey, Dunnett, or Games-Howell post hoc tests were used to compare multiple data sets. Correlation of data sets was assessed by a two-tailed Pearson test. Differences between data sets were considered significant whenever *p* values were inferior to 0.05.

### Reporting summary

Additional information on the design of this study is available in the Nature Research Reporting Summary of this article.

## Supplementary information

Supplementary Information

Reporting Summary

Supplementary Data

## Data Availability

All data supporting the findings of this study are available within the article and its supplementary information files or from the corresponding author upon request. RNA-sequencing data and annotated circRNAs are available under the Gene Expression Omnibus (GEO) accession code GSE134699. The source data underlying Supplementary Fig. 2 (ab1 raw sequencing files) are provided as a Supplementary Data compressed archive file. Source data are provided with this paper.

## Source data

Source Data
