# Supplementary material for: A circular RNA generated from an intron of the insulin gene controls insulin secretion
Source: Nat Commun. 2020 Nov 5;11:5611. doi: 10.1038/s41467-020-19381-w (PMC7644714; doi:10.1038/s41467-020-19381-w)
Supplement: Supplementary file 1 — Supplementary Information [file 41467_2020_19381_MOESM1_ESM.pdf]

Supplementary figures and tables of the manuscript "A circular RNA generated from an intron of the insulin gene controls insulin secretion"

Supplementary figures for:

A circular RNA generated from an intron of the insulin gene controls insulin secretion

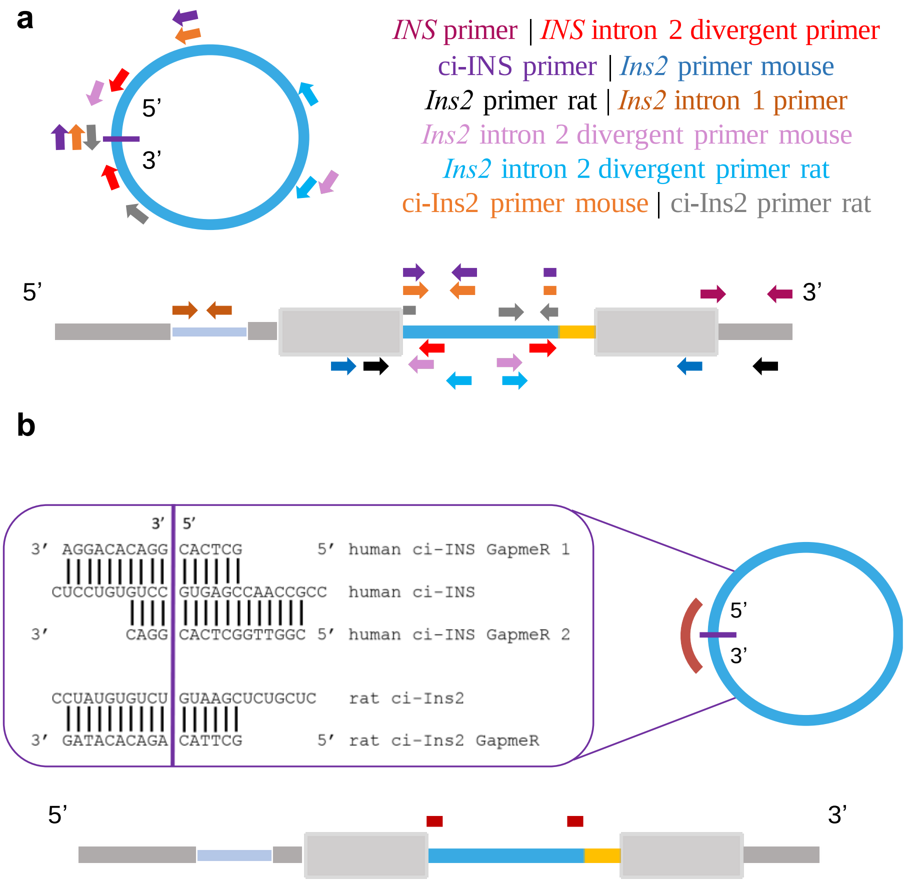

Supplementary Fig. 1 | ci-Ins2/*Ins2* and ci-INS/*INS* primer and GapmeR binding sites. Schematic representation of the binding sites of the corresponding (**a**) primers and (**b**) GapmeRs on the *Ins2/INS* and ci-Ins2/ci-INS sequences. The following color code has been used for the represented sequences: UTR Exons, CDS Exons, Intron 1, ci-Ins2/ci-INS sequence, Intron 2 lariat tail, GapmeR.

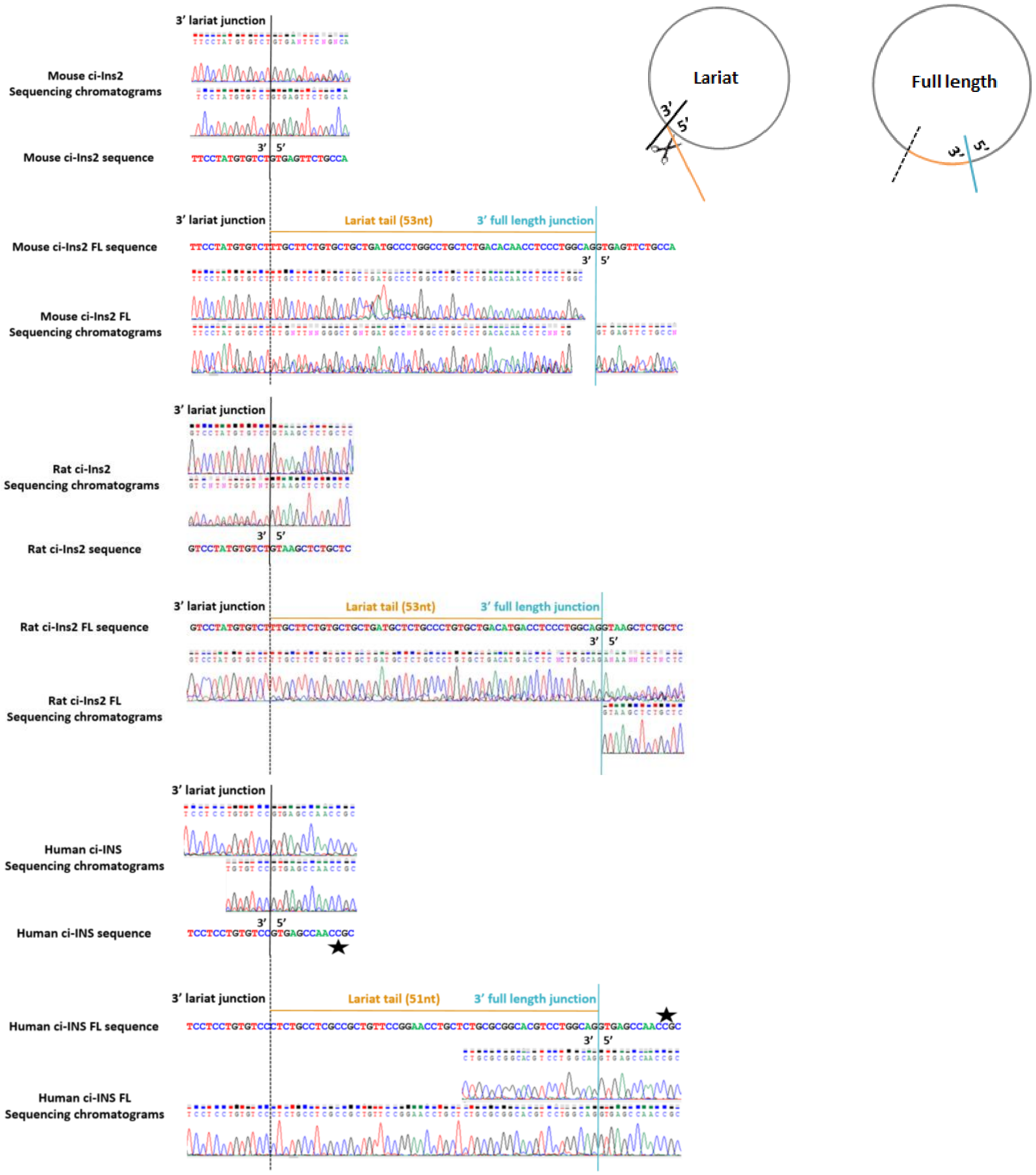

Supplementary Fig. 2 | Lariat- and full length-derived ci-Ins2 and ci-INS sequences. Chromatograms of two independent Sanger sequencings as well as genomic sequences of intron 2 lariat- and full length (FL)-derived mouse and rat ci-Ins2 and of human ci-INS. *T in genomic sequence. The scheme represents the lariat- and FL-derived ci-Ins2/ci-INS sequences. The lariat tail, which is trimmed during lariat splicing but retained in the full-length sequence, is shown in orange; the canonical intron junction is displayed in blue.

Supplementary Table 1 | Predicted circRNAs derived from key β-cell genes.

* Total number of back splice spanning reads.

**circRNA/mRNA ratio indicates the average ratio of circRNA expression relative to the linear expression of the host gene, calculated as: 2*BSJ-reads / (2*BSJ-reads + linear-reads). 1 =100% circular, 0 = 0% circular.

circRNA_parent gene	Chromosome	Start	End	Junction reads*	Antisense gene	circRNA type	circRNA/mRNA ratio**
circ_08584_Ins1	chr19	52264648	52264853	2996143	Ins1	exon	3.06E-06
circ_25207_Ins1	chr19	52264807	52264991	2928280	Ins1	exon	1.37E-06
circ_03986_Ins2	chr7	142678859	142679346	52733	.	intron	3.93E-04
circ_13543_Ins2	chr7	142678903	142679346	42011	.	intron	1.35E-04
circ_11718_Ins2	chr7	142678915	142679346	39218	.	intron	2.55E-04
circ_07027_Chga	chr12	102562758	102563073	308983	.	exon	5.18E-05
circ_02192_Chga	chr12	102561344	102563073	170443	.	exon	1.40E-04
circ_27528_Chga	chr12	102564794	102564964	2	Chga	exon	1.21E-05
circ_27531_Chga	chr12	102562800	102563073	2	.	exon	1.14E-05
circ_12242_Chga	chr12	102558465	102563073	2	.	exon	1.41E-05
circ_24998_Chgb	chr2	132793357	132793683	31570	.	exon	1.27E-04
circ_09621_Gck	chr11	5906476	5906778	2313	.	exon	6.87E-03
circ_10818_Gck	chr11	5906476	5911024	1985	.	exon	3.01E-03
circ_06464_Glp1r	chr17	30924500	30931287	9960	.	exon	8.03E-04
circ_07242_Glp1r	chr17	30918879	30924654	5813	.	exon	1.12E-03
circ_00791_Glp1r	chr17	30916216	30919462	4937	.	intron	1.77E-02
circ_26918_Pcsk1	chr13	75125849	75127943	15799	.	exon	2.53E-04
circ_11409_Pcsk1	chr13	75097900	75112754	13723	.	exon	5.96E-04
circ_00025_Pcsk2	chr2	143687563	143696694	91618	.	exon	1.38E-02
circ_07537_Pcsk2	chr2	143749124	143801231	86958	Pcsk2os2	exon	8.46E-05
circ_11959_Pcsk2	chr2	143749124	143784703	86274	Pcsk2os2	exon	4.64E-05
circ_04004_Pcsk2	chr2	143687563	143690435	86141	.	exon	2.55E-04
circ_04699_Pcsk2	chr2	143687563	143773989	85512	Pcsk2os2	exon	1.16E-04
circ_00541_Pcsk2	chr2	143687563	143749201	85226	Pcsk2os2	exon	1.08E-03
circ_12809_Pcsk2	chr2	143784527	143813532	77545	Pcsk2os2	exon	7.74E-05
circ_08211_Pcsk2	chr2	143784527	143801231	76932	Pcsk2os2	exon	4.71E-05
circ_03036_Pcsk2	chr2	143773900	143801231	75521	Pcsk2os2	exon	2.43E-04
circ_09427_Pcsk2	chr2	143793088	143801231	68100	.	exon	8.79E-05
circ_24972_Pcsk2	chr2	143801003	143813532	65197	.	exon	6.13E-05
circ_19800_Pcsk2	chr2	143773900	143784747	40097	Pcsk2os2	intron	1.50E-04
circ_24977_Pcsk2	chr2	143609324	143610894	93	.	intron	4.12E-02
circ_06440_Pcsk2	chr2	143687563	143705295	6	.	intron	2.43E-04
circ_12808_Pcsk2	chr2	143793088	143813532	5	.	exon	1.15E-04
circ_09429_Pcsk2	chr2	143571399	143573547	4	.	intron	1.57E-04
circ_24973_Pcsk2	chr2	143784527	143796044	2	Pcsk2os2	exon	4.48E-05
circ_15302_Pcsk2	chr2	143795943	143801231	2	.	exon	4.50E-05
circ_14161_Slc30a8	chr15	52295838	52321852	36792	.	exon	1.09E-04
circ_03692_Slc30a8	chr15	52317274	52325710	25047	.	exon	6.31E-04
circ_08283_Slc30a8	chr15	52321698	52322586	12384	.	intron	7.08E-04
circ_12123_Slc30a8	chr15	52316247	52325710	2	.	intron	2.56E-04

Supplementary Table 2 | Relative number of copies in rodent islet cells.

circRNA/gene	Nb of copies (per ng of RNA)
ci-Ins2	4.71E+02
ciRS-7	2.24E+01
circHIPK3	2.73E+03
Ins2	2.62E+06
Rapgef4	5.28E+02
Syt7	2.40E+02
Unc13a	1.36E+03

Supplementary Table 3 | Predicted binding sites of microRNAs in ci-Ins2 and ci-INS sequences.
Lariat ci-Ins2 and ci-INS sequences are shown in the Fig. 1b sheet of the Excel Source Data file.

Sequence	Start	End	miRNA	Seed match
rat ci-Ins2	202	208	miR-100-5p	ACGGGT
human ci-INS	17	23	miR-103-3p	TGCTGC
human ci-INS	117	123	miR-106b-5p	CACTTT
human ci-INS	17	23	miR-107-3p	TGCTGC
human ci-INS	361	367	miR-10a-5p	CAGGGT
human ci-INS	361	367	miR-10b-5p	CAGGGT
mouse ci-Ins2	181	187	miR-124-3p	TGCCTT
human ci-INS	414	420	miR-1249-3p	AGGGCG
human ci-INS	530	536	miR-125a-3p	CACCTG
mouse ci-Ins2	352	358	miR-125a-3p	CACCTG
rat ci-Ins2	400	406	miR-125a-5p	TCAGGG
rat ci-Ins2	400	406	miR-125b-5p	TCAGGG
human ci-INS	547	553	miR-128-3p	ACTGTG
human ci-INS	703	709	miR-128-3p	ACTGTG
human ci-INS	717	723	miR-128-3p	ACTGTG
human ci-INS	682	688	miR-129-2-3p	AGGGCT
human ci-INS	115	121	miR-130a-3p	TGCACT
rat ci-Ins2	254	260	miR-130a-3p	TGCACT
mouse ci-Ins2	267	273	miR-130a-3p	TGCACT
human ci-INS	115	121	miR-130b-3p	TGCACT
rat ci-Ins2	254	260	miR-130b-3p	TGCACT
mouse ci-Ins2	267	273	miR-130b-3p	TGCACT
rat ci-Ins2	334	340	miR-135a-5p	AGCCAT
human ci-INS	708	714	miR-139-3p	GTCTCC
rat ci-Ins2	187	193	miR-139-3p	GTCTCC
human ci-INS	602	608	miR-139-5p	CTGTAG
human ci-INS	322	328	miR-141-3p	AGTGTT
human ci-INS	490	496	miR-141-5p	GAAGAT
mouse ci-Ins2	277	283	miR-141-5p	GAAGAT
rat ci-Ins2	425	431	miR-145a-5p	ACTGGA
mouse ci-Ins2	414	420	miR-145a-5p	ACTGGA
human ci-INS	134	140	miR-146a-5p	GTTCTC
human ci-INS	134	140	miR-146b-5p	GTTCTC
human ci-INS	701	707	miR-148a-3p	GCACTG
rat ci-Ins2	250	256	miR-148a-3p	GCACTG
rat ci-Ins2	255	261	miR-148a-3p	GCACTG
mouse ci-Ins2	263	269	miR-148a-3p	GCACTG
mouse ci-Ins2	268	274	miR-148a-3p	GCACTG
human ci-INS	701	707	miR-148b-3p	GCACTG
rat ci-Ins2	250	256	miR-148b-3p	GCACTG
rat ci-Ins2	255	261	miR-148b-3p	GCACTG
mouse ci-Ins2	263	269	miR-148b-3p	GCACTG
mouse ci-Ins2	268	274	miR-148b-3p	GCACTG
human ci-INS	668	674	miR-150-5p	TGGGAG
human ci-INS	701	707	miR-152-3p	GCACTG
rat ci-Ins2	250	256	miR-152-3p	GCACTG

rat ci-Ins2	255	261	miR-152-3p	GCACTG
mouse ci-Ins2	263	269	miR-152-3p	GCACTG
mouse ci-Ins2	268	274	miR-152-3p	GCACTG
human ci-INS	638	644	miR-154-5p	TAACCT
mouse ci-Ins2	355	361	miR-17-3p	CTGCAG
human ci-INS	117	123	miR-17-5p	CACTTT
rat ci-Ins2	265	271	miR-188-5p	AGGGAT
rat ci-Ins2	55	61	miR-1894-3p	CCCTTG
rat ci-Ins2	344	350	miR-1894-3p	CCCTTG
human ci-INS	74	80	miR-1904	GCAGAA
human ci-INS	17	23	miR-1906	TGCTGC
human ci-INS	322	328	miR-200a-3p	AGTGTT
human ci-INS	117	123	miR-20a-5p	CACTTT
human ci-INS	117	123	miR-20b-5p	CACTTT
rat ci-Ins2	1	7	miR-21a-5p	TAAGCT
rat ci-Ins2	257	263	miR-223-3p	ACTGAC
mouse ci-Ins2	270	276	miR-223-3p	ACTGAC
human ci-INS	466	472	miR-22-3p	GCAGCT
rat ci-Ins2	129	135	miR-22-3p	GCAGCT
mouse ci-Ins2	154	160	miR-22-3p	GCAGCT
mouse ci-Ins2	217	223	miR-23a-3p	ATGTGA
mouse ci-Ins2	217	223	miR-23b-3p	ATGTGA
human ci-INS	1	7	miR-24-3p	TGAGCC
human ci-INS	548	554	miR-27a-3p	CTGTGA
human ci-INS	548	554	miR-27b-3p	CTGTGA
human ci-INS	48	54	miR-28a-5p	GCTCCT
human ci-INS	245	251	miR-28a-5p	GCTCCT
rat ci-Ins2	9	15	miR-28a-5p	GCTCCT
rat ci-Ins2	415	421	miR-28a-5p	GCTCCT
human ci-INS	116	122	miR-291a-3p	GCACTT
human ci-INS	303	309	miR-291a-5p	TTTGAT
mouse ci-Ins2	250	256	miR-296-5p	GGGCCC
rat ci-Ins2	5	11	miR-298-5p	CTCTGC
rat ci-Ins2	398	404	miR-29a-5p	AATCAG
rat ci-Ins2	103	109	miR-29b-1-5p	AACCAG
mouse ci-Ins2	102	108	miR-29b-1-5p	AACCAG
mouse ci-Ins2	48	54	miR-300-5p	TCTTCA
human ci-INS	115	121	miR-301a-3p	TGCACT
rat ci-Ins2	254	260	miR-301a-3p	TGCACT
mouse ci-Ins2	267	273	miR-301a-3p	TGCACT
human ci-INS	115	121	miR-301b-3p	TGCACT
rat ci-Ins2	254	260	miR-301b-3p	TGCACT
mouse ci-Ins2	267	273	miR-301b-3p	TGCACT
rat ci-Ins2	22	28	miR-30b-3p	ATCCCA
mouse ci-Ins2	227	233	miR-30b-3p	ATCCCA
human ci-INS	58	64	miR-30c-1-3p	CTCCCA
human ci-INS	542	548	miR-30c-1-3p	CTCCCA
human ci-INS	58	64	miR-30c-2-3p	CTCCCA
human ci-INS	542	548	miR-30c-2-3p	CTCCCA
rat ci-Ins2	346	352	miR-31-5p	CTTGCC

human ci-INS	437	443	miR-328-3p	GGGCCA
human ci-INS	103	109	miR-331-3p	CAGGGG
rat ci-Ins2	368	374	miR-331-3p	CAGGGG
rat ci-Ins2	401	407	miR-331-3p	CAGGGG
mouse ci-Ins2	358	364	miR-331-3p	CAGGGG
human ci-INS	137	143	miR-335-5p	CTCTTG
human ci-INS	495	501	miR-338-3p	TGCTGG
human ci-INS	40	46	miR-342-5p	CACCCC
human ci-INS	394	400	miR-342-5p	CACCCC
mouse ci-Ins2	224	230	miR-344-3p	TAGATC
human ci-INS	108	114	miR-345-5p	GGTCAG
human ci-INS	8	14	miR-34a-5p	ACTGCC
human ci-INS	8	14	miR-34b-5p	ACTGCC
human ci-INS	8	14	miR-34c-5p	ACTGCC
human ci-INS	101	107	miR-370-3p	AGCAGG
rat ci-Ins2	366	372	miR-370-3p	AGCAGG
rat ci-Ins2	436	442	miR-376c-3p	CTATGT
mouse ci-Ins2	425	431	miR-376c-3p	CTATGT
human ci-INS	646	652	miR-378a-3p	GTCCAG
human ci-INS	478	484	miR-378a-5p	TCAGGA
human ci-INS	262	268	miR-423-5p	CCCCTC
rat ci-Ins2	311	317	miR-433-3p	TCATGA
rat ci-Ins2	115	121	miR-455-5p	GCACAT
rat ci-Ins2	281	287	miR-466i-3p	TGTGTA
human ci-INS	190	196	miR-484	AGCCTG
rat ci-Ins2	388	394	miR-484	AGCCTG
rat ci-Ins2	49	55	miR-488-3p	CTTTCA
human ci-INS	327	333	miR-495-3p	TTTGTT
human ci-INS	540	546	miR-505-5p	GGCTCC
human ci-INS	668	674	miR-532-3p	TGGGAG
mouse ci-Ins2	335	341	miR-532-5p	AGGCAT
human ci-INS	289	295	miR-539-5p	TTTCTC
human ci-INS	218	224	miR-542-5p	CCCCGA
human ci-INS	156	162	miR-668-3p	AGTGAC
rat ci-Ins2	319	325	miR-668-3p	AGTGAC
mouse ci-Ins2	320	326	miR-668-3p	AGTGAC
mouse ci-Ins2	105	111	miR-670-5p	CAGGGA
human ci-INS	196	202	miR-676-3p	AGGACG
human ci-INS	220	226	miR-678	CCGAGA
rat ci-Ins2	278	284	miR-697	GGATGT
human ci-INS	289	295	miR-706	TTTCTC
human ci-INS	380	386	miR-709	TGCCTC
rat ci-Ins2	348	354	miR-709	TGCCTC
human ci-INS	572	578	miR-712-5p	GAAGGA
rat ci-Ins2	163	169	miR-712-5p	GAAGGA
human ci-INS	115	121	miR-721	TGCACT
rat ci-Ins2	254	260	miR-721	TGCACT
mouse ci-Ins2	267	273	miR-721	TGCACT
human ci-INS	277	283	miR-744-5p	CCCCGC
human ci-INS	215	221	miR-762	CAGCCC

human ci-INS	282	288	miR-762	CAGCCC
human ci-INS	649	655	miR-762	CAGCCC
human ci-INS	327	333	miR-7a-1-3p	TTTGTT
human ci-INS	638	644	miR-872-5p	TAACCT
human ci-INS	195	201	miR-877-3p	GAGGAC
human ci-INS	117	123	miR-93-5p	CACTTT
rat ci-Ins2	202	208	miR-99a-5p	ACGGGT
rat ci-Ins2	202	208	miR-99b-5p	ACGGGT

Supplementary Table 4 | microRNAs targeting genes differentially expressed after ci-Ins2 or ci-INS inhibition.

The analyzed genes are shown in Fig. 6

The hypergeometric test *p* value was obtained by using the Enrichr webserver.

Term	*p* value	Adjusted *p* value	Z-score	Combined score	Genes
hsa-miR-1273c	0.0261	0.20	-0.55	2.01	SLC12A5;SYT7
hsa-miR-1306	0.0350	0.22	-0.64	2.14	MIDN;SYT7
hsa-miR-139-3p	0.0261	0.20	-1.23	4.48	SLC12A5;GIPR;SYT7
hsa-miR-184	0.0153	0.15	-1.22	5.08	SLC12A5;UNC13A;SIK1
hsa-miR-1908	0.0015	0.10	-3.21	20.87	MIDN;SLC12A5;UNC13A;SIK1;CACNA1D;SYT7
hsa-miR-3135	0.0360	0.22	-2.39	7.95	SYT4;PCLO;SIK1;MICU1
hsa-miR-3141	0.0259	0.20	-1.30	4.75	MIDN;UNC13A;CACNA1D
hsa-miR-3178	0.0195	0.16	-1.24	4.89	MIDN;SLC12A5;UNC13A
hsa-miR-3180	0.0035	0.10	-2.36	13.36	MIDN;SLC12A5;SIK1;MICU1;SYT7
hsa-miR-3180-3p	0.0035	0.10	-2.35	13.33	MIDN;SLC12A5;SIK1;MICU1;SYT7
hsa-miR-3196	0.0035	0.10	-2.35	13.30	MIDN;SLC12A5;SIK1;MICU1;SYT7
hsa-miR-3681	0.0315	0.22	-2.29	7.92	SYT4;PCLO;PLD1;RAPGEF4
hsa-miR-3689a-5p	0.0095	0.12	-2.95	13.72	SYT4;SLC12A5;PCLO;CACNA1D;MICU1
hsa-miR-3689b	0.0095	0.12	-2.94	13.70	SYT4;SLC12A5;PCLO;CACNA1D;MICU1
hsa-miR-3689e	0.0095	0.12	-2.93	13.66	SYT4;SLC12A5;PCLO;CACNA1D;MICU1
hsa-miR-3689f	0.0095	0.12	-2.93	13.63	SYT4;SLC12A5;PCLO;CACNA1D;MICU1
hsa-miR-383	0.0115	0.13	-2.96	13.20	RIMS2;PCLO;UNC13A;PLD1;SYT7
hsa-miR-3960	0.0475	0.22	-0.44	1.34	MIDN;SLC12A5
hsa-miR-4258	0.0015	0.10	-2.01	13.14	RIMS2;MIDN;UNC13A;GIPR;SYT7
hsa-miR-4259	0.0119	0.13	-3.07	13.58	RIMS2;PCLO;UNC13A;CACNA1D;SYT7
hsa-miR-4280	0.0286	0.21	-2.23	7.93	RIMS2;PCLO;UNC13A;CACNA1D
hsa-miR-4317	0.0436	0.22	-2.49	7.79	SLC12A5;PCLO;SIK1;CACNA1D
hsa-miR-4508	0.0078	0.11	-1.79	8.67	MIDN;SLC12A5;UNC13A;SYT7
hsa-miR-4535	0.0166	0.16	-2.03	8.32	RIMS2;SLC12A5;PCLO;SIK1
hsa-miR-4632	0.0031	0.10	-1.63	9.41	MIDN;SLC12A5;SIK1;SYT7
hsa-miR-4640-3p	0.0405	0.22	-2.52	8.09	MIDN;SLC12A5;GIPR;SYT7
hsa-miR-4655-5p	0.0497	0.22	-0.30	0.90	MIDN;SLC12A5
hsa-miR-4665-3p	0.0285	0.21	-0.58	2.08	SLC12A5;UNC13A

hsa-miR-4694-5p	0.0478	0.22	-2.60	7.90	RIMS2;SLC12A5;UNC13A;SIK1
hsa-miR-4707-5p	0.0474	0.22	-1.25	3.82	SLC12A5;UNC13A;SYT7
hsa-miR-4715-5p	0.0290	0.21	-2.34	8.27	RIMS2;PCLO;UNC13A;GIPR
hsa-miR-4727-3p	0.0266	0.20	-2.19	7.96	PCLO;CACNA1D;PLD1;RAPGEF4
hsa-miR-4737	0.0046	0.11	-1.24	6.69	MIDN;CACNA1D;MICU1
hsa-miR-4759	0.0368	0.22	-2.34	7.74	RIMS2;PCLO;PLD1;SYT7
hsa-miR-4776-5p	0.0236	0.19	-2.20	8.24	RIMS2;SLC12A5;PCLO;SIK1
hsa-miR-4787-5p	0.0319	0.22	-1.34	4.62	MIDN;UNC13A;SYT7
hsa-miR-483-5p	0.0444	0.22	-1.38	4.30	SLC12A5;UNC13A;SYT7
hsa-miR-492	0.0096	0.12	-2.96	13.78	MIDN;SLC12A5;PCLO;CACNA1D;SYT7
hsa-miR-501-3p	0.0479	0.22	-2.54	7.73	RIMS2;UNC13A;GIPR;SYT7
hsa-miR-502-3p	0.0479	0.22	-2.54	7.71	RIMS2;UNC13A;GIPR;SYT7
hsa-miR-503	0.0454	0.22	-2.50	7.73	MIDN;SLC12A5;UNC13A;SIK1
hsa-miR-518a-3p	0.0471	0.22	-0.49	1.49	SLC12A5;UNC13A
hsa-miR-518b	0.0471	0.22	-0.48	1.47	SLC12A5;UNC13A
hsa-miR-518c	0.0471	0.22	-0.47	1.44	SLC12A5;UNC13A
hsa-miR-518d-3p	0.0471	0.22	-0.46	1.41	SLC12A5;UNC13A
hsa-miR-518f	0.0471	0.22	-0.45	1.39	SLC12A5;UNC13A
hsa-miR-524-3p	0.0462	0.22	-0.43	1.33	UNC13A;SYT7
hsa-miR-525-3p	0.0462	0.22	-0.42	1.30	UNC13A;SYT7
hsa-miR-556-5p	0.0428	0.22	-2.43	7.64	SLC12A5;PCLO;SIK1;MICU1
hsa-miR-566	0.0258	0.20	-1.33	4.88	SLC12A5;UNC13A;SYT7
hsa-miR-602	0.0034	0.10	-1.62	9.18	MIDN;SLC12A5;GIPR;MICU1
hsa-miR-639	0.0069	0.11	-1.19	5.93	MIDN;CACNA1D;SYT7
hsa-miR-663	0.0015	0.10	-3.20	20.83	MIDN;SLC12A5;UNC13A;SIK1;CACNA1D;SYT7
hsa-miR-718	0.0401	0.22	-0.49	1.57	SLC12A5;GIPR
hsa-miR-720	0.0363	0.22	-1.34	4.45	MIDN;GIPR;CACNA1D
hsa-miR-744	0.0019	0.10	-2.18	13.66	MIDN;SLC12A5;UNC13A;CACNA1D;SYT7
hsa-miR-934	0.0005	0.09	-2.47	18.78	SYT4;SLC12A5;PCLO;SIK1;PLD1;SYT7
hsa-miR-937	0.0002	0.05	-1.21	10.41	SLC12A5;UNC13A;SYT7
mmu-miR-1193-5p	0.0155	0.15	-1.98	8.24	MIDN;UNC13A;SIK1;SYT7
mmu-miR-122	0.0494	0.22	-2.51	7.56	SLC12A5;UNC13A;MICU1;SYT7
mmu-miR-1298	0.0072	0.11	-2.77	13.69	RIMS2;PCLO;ATP1B3;ATP1A1;RAPGEF4

mmu-miR-133a	0.0495	0.22	-2.43	7.31	RIMS2;PCLO;UNC13A;PLD1
mmu-miR-133b	0.0495	0.22	-2.43	7.29	RIMS2;PCLO;UNC13A;PLD1
mmu-miR-137	0.0126	0.14	-3.14	13.73	RIMS2;SLC12A5;PCLO;SIK1;CACNA1D
mmu-miR-155	0.0056	0.11	-2.57	13.35	RIMS2;MIDN;PCLO;PLD1;RAPGEF4
mmu-miR-183	0.0099	0.12	-2.81	12.98	SLC12A5;PCLO;SIK1;SYT7;RAPGEF4
mmu-miR-1839-3p	0.0073	0.11	-1.79	8.82	RIMS2;PCLO;GIPR;SIK1
mmu-miR-188-5p	0.0053	0.11	-2.57	13.45	RIMS2;UNC13A;ATP1B3;CACNA1D;PLD1
mmu-miR-1895	0.0135	0.14	-1.17	5.06	RIMS2;SIK1;RAPGEF4
mmu-miR-18a	0.0064	0.11	-2.66	13.43	RIMS2;PCLO;MICU1;SYT7;RAPGEF4
mmu-miR-18b	0.0064	0.11	-2.65	13.40	RIMS2;PCLO;MICU1;SYT7;RAPGEF4
mmu-miR-192	0.0029	0.10	-2.35	13.77	PCLO;UNC13A;GIPR;SIK1;SYT7
mmu-miR-1941-3p	0.0071	0.11	-2.77	13.74	RIMS2;SYT4;CACNA1D;PLD1;RAPGEF4
mmu-miR-1946a	0.0083	0.12	-1.19	5.69	SLC12A5;CACNA1D;SYT7
mmu-miR-1951	0.0107	0.12	-1.73	7.86	PCLO;PLD1;SYT7;RAPGEF4
mmu-miR-1957	0.0183	0.16	-1.98	7.92	RIMS2;SLC12A5;PCLO;SYT7
mmu-miR-202-5p	0.0459	0.22	-2.52	7.76	PCLO;UNC13A;SIK1;RAPGEF4
mmu-miR-210	0.0498	0.22	-0.38	1.13	RIMS2;PLD1
mmu-miR-2137	0.0042	0.11	-0.73	4.02	RIMS2;SLC12A5
mmu-miR-215	0.0029	0.10	-2.35	13.74	PCLO;UNC13A;GIPR;SIK1;SYT7
mmu-miR-219-3p	0.0026	0.10	-2.22	13.17	SYT4;MIDN;PCLO;UNC13A;SYT7
mmu-miR-25	0.0366	0.22	-2.44	8.07	RIMS2;SLC12A5;PCLO;SIK1
mmu-miR-296-5p	0.0011	0.10	-3.12	21.10	MIDN;SLC12A5;UNC13A;ATP1B3;PLD1;SYT7
mmu-miR-3070a	0.0060	0.11	-2.71	13.85	RIMS2;ATP1A1;MICU1;SYT7;RAPGEF4
mmu-miR-3070b-3p	0.0060	0.11	-2.70	13.82	RIMS2;ATP1A1;MICU1;SYT7;RAPGEF4
mmu-miR-3073-3p	0.0372	0.22	-2.30	7.58	SLC12A5;PCLO;ATP1B3;PLD1
mmu-miR-3074-1-3p	0.0056	0.11	-2.58	13.35	MIDN;SLC12A5;PCLO;PLD1;RAPGEF4
mmu-miR-3080-3p	0.0032	0.10	-1.61	9.26	SYT4;SLC12A5;UNC13A;SYT7
mmu-miR-3092	0.0347	0.22	-2.44	8.20	SLC12A5;PCLO;UNC13A;SYT7
mmu-miR-3093-5p	0.0035	0.10	-2.43	13.74	MIDN;UNC13A;GIPR;CACNA1D;SYT7
mmu-miR-3101	0.0140	0.15	-1.90	8.10	RIMS2;ATP1B3;CACNA1D;PLD1
mmu-miR-3102-3p,2	0.0436	0.22	-2.46	7.70	RIMS2;SYT4;SLC12A5;SIK1
mmu-miR-3103	0.0300	0.22	-2.25	7.88	SYT4;SLC12A5;PCLO;CACNA1D
mmu-miR-32	0.0366	0.22	-2.43	8.04	RIMS2;SLC12A5;PCLO;SIK1

mmu-miR-340-3p	0.0076	0.11	-1.17	5.72	MIDN;SLC12A5;CACNA1D
mmu-miR-363-3p	0.0366	0.22	-2.43	8.02	RIMS2;SLC12A5;PCLO;SIK1
mmu-miR-367	0.0366	0.22	-2.42	8.00	RIMS2;SLC12A5;PCLO;SIK1
mmu-miR-379	0.0155	0.15	-1.97	8.21	MIDN;UNC13A;SIK1;SYT7
mmu-miR-3963	0.0373	0.22	-1.33	4.37	RIMS2;PCLO;ATP1B3
mmu-miR-466b-5p	0.0438	0.22	-2.46	7.71	RIMS2;PCLO;CACNA1D;RAPGEF4
mmu-miR-466c-5p	0.0438	0.22	-2.46	7.69	RIMS2;PCLO;CACNA1D;RAPGEF4
mmu-miR-466o-5p	0.0438	0.22	-2.45	7.67	RIMS2;PCLO;CACNA1D;RAPGEF4
mmu-miR-471-5p	0.0150	0.15	-0.50	2.09	PCLO;SIK1
mmu-miR-500	0.0182	0.16	-2.05	8.22	MIDN;PCLO;CACNA1D;PLD1
mmu-miR-501-3p	0.0182	0.16	-2.05	8.20	MIDN;PCLO;CACNA1D;PLD1
mmu-miR-503	0.0326	0.22	-2.23	7.64	RIMS2;MIDN;UNC13A;SIK1
mmu-miR-5103	0.0416	0.22	-0.55	1.75	MIDN;UNC13A
mmu-miR-5128	0.0010	0.10	-2.00	13.85	RIMS2;SYT4;MIDN;SLC12A5;UNC13A
mmu-miR-5130	0.0103	0.12	-1.73	7.93	RIMS2;MIDN;SLC12A5;SYT7
mmu-miR-5135	0.0112	0.13	-1.86	8.37	SYT4;ATP1B3;PLD1;RAPGEF4
mmu-miR-615-5p	0.0001	0.05	-2.88	25.74	RIMS2;MIDN;UNC13A;GIPR;ATP1B3;MICU1;SYT7
mmu-miR-653	0.0345	0.22	-2.39	8.04	RIMS2;SYT4;PCLO;PLD1
mmu-miR-669a-3-3p	0.0052	0.11	-2.50	13.18	RIMS2;SYT4;SLC12A5;PCLO;SIK1
mmu-miR-669a-3p	0.0052	0.11	-2.50	13.14	RIMS2;SYT4;SLC12A5;PCLO;SIK1
mmu-miR-669a-5p	0.0190	0.16	-2.02	8.01	RIMS2;SLC12A5;PCLO;RAPGEF4
mmu-miR-669f-5p	0.0190	0.16	-2.01	7.98	RIMS2;SLC12A5;PCLO;RAPGEF4
mmu-miR-669l	0.0190	0.16	-2.01	7.95	RIMS2;SLC12A5;PCLO;RAPGEF4
mmu-miR-669o-3p	0.0052	0.11	-2.49	13.11	RIMS2;SYT4;SLC12A5;PCLO;SIK1
mmu-miR-669p	0.0190	0.16	-2.00	7.93	RIMS2;SLC12A5;PCLO;RAPGEF4
mmu-miR-671-3p	0.0267	0.20	-0.56	2.01	UNC13A;GIPR
mmu-miR-707	0.0075	0.11	-2.76	13.52	RIMS2;SYT4;PCLO;SIK1;CACNA1D
mmu-miR-720	0.0363	0.22	-0.53	1.76	MIDN;PCLO
mmu-miR-770-3p	0.0442	0.22	-2.55	7.96	SLC12A5;SIK1;PLD1;SYT7
mmu-miR-880	0.0099	0.12	-2.93	13.52	SYT4;SLC12A5;PCLO;PLD1;RAPGEF4
mmu-miR-92a	0.0366	0.22	-2.41	7.98	RIMS2;SLC12A5;PCLO;SIK1
mmu-miR-92b	0.0366	0.22	-2.41	7.96	RIMS2;SLC12A5;PCLO;SIK1

Supplementary Table 5 | Predicted binding sites of transcription factors in ci-Ins2 and ci-INS sequences.

Lariat ci-Ins2 and ci-INS sequences are shown in the Fig. 1b sheet of the Excel Source Data file.

The sequence match *p* value was calculated with FIMO's dynamic programming algorithm.

motif_id	motif_alt_id	sequence_name	start	end	*p* _value	matched_sequence
MA0816.1	Ascl2	mouse ci-Ins2	154	163	7.64e-06	ggcagctgcc
MA0461.2	Atoh1	rat ci-Ins2	137	146	6.83e-05	gacatatgta
MA0461.2	Atoh1	rat ci-Ins2	299	308	6.44e-05	cccatatgtc
MA0461.2	Atoh1	mouse ci-Ins2	139	148	6.83e-05	gacatatgta
MA0591.1	Bach1::Mafk	rat ci-Ins2	357	371	5.91e-05	gtgctgacttagcag
MA0462.1	BATF::JUN	mouse ci-Ins2	165	175	2.43e-05	ggaatgagtaa
MA0607.1	Bhlha15	rat ci-Ins2	300	307	1.79e-05	ccatatgt
MA0818.1	BHLHE22	rat ci-Ins2	299	308	7.97e-05	cccatatgtc
MA0817.1	BHLHE23	rat ci-Ins2	136	147	4.87e-05	agacatatgtaa
MA0817.1	BHLHE23	mouse ci-Ins2	138	149	4.87e-05	agacatatgtaa
MA0139.1	CTCF	human ci-INS	72	90	1.93e-06	tgggcagaagggggcagga
MA0139.1	CTCF	rat ci-Ins2	103	121	5.67e-06	taaccagtggggggcacat
MA0019.1	Ddit3::Cebpa	mouse ci-Ins2	338	349	1.06e-05	gcatgcaacccc
MA0470.1	E2F4	human ci-INS	565	575	5.3e-05	gggcgggggaa
MA0470.1	E2F4	mouse ci-Ins2	370	380	8.34e-05	gggcgagaaaa
MA0162.2	EGR1	human ci-INS	27	40	1.78e-05	tggccgcccccagc
MA0162.2	EGR1	human ci-INS	37	50	1.42e-05	cagccaccccctgc
MA0641.1	ELF4	mouse ci-Ins2	103	114	9.67e-05	aaccagggagtg
MA0760.1	ERF	rat ci-Ins2	276	285	6.28e-05	acaggatgtg
MA0474.2	ERG	rat ci-Ins2	276	285	8.52e-05	acaggatgtg
MA0258.2	ESR2	human ci-INS	356	370	7.12e-05	gggtcacagggtgcc
MA0098.3	ETS1	rat ci-Ins2	276	285	5.48e-05	acaggatgtg
MA0762.1	ETV2	rat ci-Ins2	275	285	8.25e-05	aacaggatgtg
MA0149.1	EWSR1-FLI1	human ci-INS	564	581	3.28e-05	ggggcgggggaaggaggt
MA0820.1	FIGLA	human ci-INS	529	538	2.3e-05	atcacctgtt
MA0478.1	FOSL2	mouse ci-Ins2	166	176	9.2e-05	gaatgagtaag
MA0845.1	FOXB1	rat ci-Ins2	141	151	9.99e-07	tatgtaaacat
MA0845.1	FOXB1	mouse ci-Ins2	143	153	9.99e-07	tatgtaaacat

MA0032.2	FOXC1	rat ci-Ins2	141	151	4.51e-07	tatgtaaacat
MA0032.2	FOXC1	mouse ci-Ins2	143	153	4.51e-07	tatgtaaacat
MA0846.1	FOXC2	rat ci-Ins2	141	152	1.45e-05	tatgtaaacatg
MA0846.1	FOXC2	mouse ci-Ins2	143	154	1.45e-05	tatgtaaacatg
MA0031.1	FOXD1	rat ci-Ins2	144	151	2.18e-05	gtaaacat
MA0031.1	FOXD1	mouse ci-Ins2	146	153	2.18e-05	gtaaacat
MA0847.1	FOXD2	rat ci-Ins2	144	150	7.93e-05	gtaaaca
MA0847.1	FOXD2	mouse ci-Ins2	146	152	7.93e-05	gtaaaca
MA0613.1	FOXG1	rat ci-Ins2	144	151	4.35e-05	gtaaacat
MA0613.1	FOXG1	mouse ci-Ins2	146	153	4.35e-05	gtaaacat
MA0042.2	FOXI1	rat ci-Ins2	144	150	7.93e-05	gtaaaca
MA0042.2	FOXI1	mouse ci-Ins2	146	152	7.93e-05	gtaaaca
MA0614.1	Foxj2	rat ci-Ins2	144	151	8.79e-05	gtaaacat
MA0614.1	Foxj2	mouse ci-Ins2	146	153	8.79e-05	gtaaacat
MA0852.1	Foxk1	rat ci-Ins2	139	155	3.77e-05	catatgtaaacatggta
MA0852.1	Foxk1	mouse ci-Ins2	141	157	2.87e-05	catatgtaaacatggca
MA0033.2	FOXL1	rat ci-Ins2	144	150	7.93e-05	gtaaaca
MA0033.2	FOXL1	mouse ci-Ins2	146	152	7.93e-05	gtaaaca
MA0157.2	FOXO3	rat ci-Ins2	144	151	4.35e-05	gtaaacat
MA0157.2	FOXO3	mouse ci-Ins2	146	153	4.35e-05	gtaaacat
MA0848.1	FOXO4	rat ci-Ins2	144	150	7.93e-05	gtaaaca
MA0848.1	FOXO4	mouse ci-Ins2	146	152	7.93e-05	gtaaaca
MA0849.1	FOXO6	rat ci-Ins2	144	150	7.93e-05	gtaaaca
MA0849.1	FOXO6	mouse ci-Ins2	146	152	7.93e-05	gtaaaca
MA0593.1	FOXP2	rat ci-Ins2	142	152	8.83e-05	atgtaaacatg
MA0593.1	FOXP2	mouse ci-Ins2	144	154	8.83e-05	atgtaaacatg
MA0850.1	FOXP3	rat ci-Ins2	144	150	7.93e-05	gtaaaca
MA0850.1	FOXP3	mouse ci-Ins2	146	152	7.93e-05	gtaaaca
MA0483.1	Gfi1b	human ci-INS	184	194	2.58e-05	gaatctcagcc
MA0734.1	GLI2	human ci-INS	58	69	8.34e-05	gctcccacccag
MA0736.1	GLIS2	human ci-INS	367	380	7.88e-05	tgccccacgctgcc
MA1099.1	Hes1	human ci-INS	241	250	5.15e-05	ggcacgctcc
MA0907.1	HOXC13	rat ci-Ins2	65	75	8.19e-05	ccttgtaaatt

MA0824.1	ID4	mouse ci-Ins2	154	163	8.38e-05	ggcagctgcc
MA0155.1	INSM1	mouse ci-Ins2	123	134	1.83e-05	tttctgggggaa
MA0489.1	JUN(var.2)	mouse ci-Ins2	163	176	7.73e-05	caggaatgagtaag
MA0490.1	JUNB	mouse ci-Ins2	166	176	8.15e-05	gaatgagtaag
MA0493.1	Klf1	human ci-INS	609	619	1.52e-05	gtccacaccca
MA0039.2	Klf4	human ci-INS	415	424	4.2e-06	agggcgtggc
MA0039.2	Klf4	human ci-INS	564	573	1.91e-05	ggggcggggg
MA0599.1	KLF5	human ci-INS	59	68	8.04e-05	ctcccaccca
MA0599.1	KLF5	human ci-INS	246	255	4.19e-05	gctcctccct
MA0599.1	KLF5	human ci-INS	292	301	7.92e-05	tctccaccct
MA0599.1	KLF5	human ci-INS	610	619	3.95e-05	tccacaccca
MA0768.1	LEF1	human ci-INS	226	240	7.8e-05	atacatcagagggtg
MA0705.1	Lhx8	rat ci-Ins2	397	404	8.79e-05	ctaatcag
MA0501.1	MAF::NFE2	rat ci-Ins2	360	374	9.44e-05	ctgacttagcagggg
MA0659.1	MAFG	rat ci-Ins2	354	374	1.93e-05	cctgtgctgacttagcagggg
MA0100.2	Myb	rat ci-Ins2	153	162	8.95e-05	gtagctgcca
MA0100.2	Myb	mouse ci-Ins2	155	164	6.58e-05	gcagctgcca
MA0147.2	Myc	human ci-INS	585	594	9.33e-05	acatgtgggc
MA0147.2	Myc	mouse ci-Ins2	301	310	4.12e-05	ccatgtgtcc
MA0499.1	Myod1	mouse ci-Ins2	154	166	2.2e-05	ggcagctgccagg
MA0500.1	Myog	mouse ci-Ins2	154	164	1.32e-05	ggcagctgcca
MA0057.1	MZF1(var.2)	human ci-INS	566	575	9.52e-05	ggcgggggaa
MA0668.1	NEUROD2	rat ci-Ins2	137	146	7.68e-05	gacatatgta
MA0668.1	NEUROD2	rat ci-Ins2	299	308	6.83e-05	cccatatgtc
MA0668.1	NEUROD2	mouse ci-Ins2	139	148	7.68e-05	gacatatgta
MA0623.1	Neurog1	rat ci-Ins2	299	308	4.88e-05	cccatatgtc
MA0150.2	Nfe2l2	rat ci-Ins2	356	370	5.27e-05	tgtgctgacttagca
MA0048.2	NHLH1	mouse ci-Ins2	154	163	2.38e-05	ggcagctgcc
MA0494.1	Nr1h3::Rxra	human ci-INS	349	367	9.5e-05	tgacctggggtcacagggt
MA0504.1	NR2C2	human ci-INS	664	678	9.07e-05	atgggtgggagtgcg
MA0504.1	NR2C2	mouse ci-Ins2	360	374	6.21e-06	aggggttaaggggcg
MA0017.2	NR2F1	human ci-INS	105	117	7.85e-05	agggggtcaggtg
MA0017.2	NR2F1	human ci-INS	353	365	1.06e-05	ctggggtcacagg

MA0677.1	Nr2f6	mouse ci-Ins2	361	374	4.95e-05	ggggttaaggggcg
MA0678.1	OLIG2	rat ci-Ins2	299	308	8.35e-05	cccatatgtc
MA0827.1	OLIG3	rat ci-Ins2	299	308	6.13e-05	cccatatgtc
MA0782.1	PKNOX1	mouse ci-Ins2	153	164	9.69e-06	tggcagctgcca
MA0783.1	PKNOX2	rat ci-Ins2	136	147	7.32e-05	agacatatgtaa
MA0783.1	PKNOX2	rat ci-Ins2	151	162	6.3e-05	tggtagctgcca
MA0783.1	PKNOX2	mouse ci-Ins2	138	149	7.32e-05	agacatatgtaa
MA0783.1	PKNOX2	mouse ci-Ins2	153	164	6.53e-06	tggcagctgcca
MA0142.1	Pou5f1::Sox2	human ci-INS	327	341	8.66e-05	ttttgttaagtaaag
MA0066.1	PPARG	human ci-INS	622	641	3.92e-05	gtgggtgaccctccctctaa
MA0508.1	PRDM1	rat ci-Ins2	375	389	9.44e-05	ataaagtgagagaaa
MA0859.1	Rarg	human ci-INS	355	370	9.39e-05	ggggtcacagggtgcc
MA0860.1	Rarg(var.2)	human ci-INS	345	361	4.8e-05	tgggtgacctggggtca
MA0101.1	REL	mouse ci-Ins2	417	426	4.65e-05	tggattttcc
MA0107.1	RELA	mouse ci-Ins2	417	426	3.58e-05	tggattttcc
MA0138.2	REST	human ci-INS	466	486	5.88e-05	ggcagctccatagtcaggaga
MA0138.2	REST	rat ci-Ins2	210	230	7.31e-05	gctaggccccaggataggtac
MA0073.1	RREB1	human ci-INS	33	52	9.51e-06	cccccagccaccccctgctc
MA0073.1	RREB1	human ci-INS	264	283	7.5e-05	ccctcaaacaaatgccccgc
MA0684.1	RUNX3	human ci-INS	309	318	6.43e-05	tgaccgcaga
MA0512.2	Rxra	human ci-INS	355	368	9.88e-05	ggggtcacagggtg
MA0512.2	Rxra	mouse ci-Ins2	361	374	2.48e-05	ggggttaaggggcg
MA0074.1	RXRA::VDR	human ci-INS	356	370	8.03e-06	gggtcacagggtgcc
MA0855.1	RXRB	human ci-INS	355	368	9.19e-05	ggggtcacagggtg
MA0855.1	RXRB	mouse ci-Ins2	361	374	1.66e-05	ggggttaaggggcg
MA0856.1	RXRG	mouse ci-Ins2	361	374	1.94e-05	ggggttaaggggcg
MA0513.1	SMAD2::SMAD3::SMAD4	human ci-INS	89	101	2.61e-05	gaggctgccaccc
MA0513.1	SMAD2::SMAD3::SMAD4	rat ci-Ins2	46	58	3.4e-05	ttgtctttcaccc
MA0513.1	SMAD2::SMAD3::SMAD4	rat ci-Ins2	326	338	2.15e-05	ttgtctcacagcc
MA0745.1	SNAI2	human ci-INS	110	118	7.34e-05	gtcaggtgc
MA0079.3	SP1	human ci-INS	246	256	1.71e-05	gctcctccctc
MA0079.3	SP1	human ci-INS	292	302	6.76e-05	tctccaccctc
MA0516.1	SP2	human ci-INS	246	260	4.34e-05	gctcctccctccact

MA0746.1	SP3	human ci-INS	610	620	6.62e-05	tccacacccag
MA0686.1	SPDEF	rat ci-Ins2	275	285	2.12e-05	aacaggatgtg
MA0687.1	SPIC	mouse ci-Ins2	165	178	4.32e-05	ggaatgagtaagaa
MA0111.1	Spz1	rat ci-Ins2	270	280	3.8e-05	atggtaacagg
MA0829.1	Srebf1(var.2)	human ci-INS	529	538	6.87e-05	atcacctgtt
MA0828.1	SREBF2(var.2)	human ci-INS	529	538	9.02e-05	atcacctgtt
MA0137.3	STAT1	rat ci-Ins2	157	167	6.87e-05	ctgccaggaag
MA0137.3	STAT1	mouse ci-Ins2	159	169	7.61e-05	ctgccaggaat
MA0144.2	STAT3	rat ci-Ins2	157	167	2.41e-05	ctgccaggaag
MA0144.2	STAT3	mouse ci-Ins2	159	169	3.41e-05	ctgccaggaat
MA0518.1	Stat4	rat ci-Ins2	157	170	7.07e-06	ctgccaggaaggag
MA0518.1	Stat4	mouse ci-Ins2	159	172	4.32e-05	ctgccaggaatgag
MA0803.1	TBX15	human ci-INS	578	585	8.34e-05	aggtggga
MA0689.1	TBX20	rat ci-Ins2	82	92	3.47e-05	taggtgtggag
MA0689.1	TBX20	mouse ci-Ins2	81	91	3.47e-05	taggtgtggag
MA0807.1	TBX5	rat ci-Ins2	83	90	7.13e-05	aggtgtgg
MA0807.1	TBX5	mouse ci-Ins2	82	89	7.13e-05	aggtgtgg
MA0521.1	Tcf12	mouse ci-Ins2	154	164	2.13e-05	ggcagctgcca
MA0522.2	TCF3	mouse ci-Ins2	351	360	9.49e-05	gccacctgca
MA0769.1	Tcf7	human ci-INS	226	237	4.25e-05	atacatcagagg
MA0810.1	TFAP2A(var.2)	human ci-INS	21	32	7.83e-05	tgcccctggccg
MA0810.1	TFAP2A(var.2)	human ci-INS	31	42	7.62e-06	cgcccccagcca
MA0810.1	TFAP2A(var.2)	human ci-INS	381	392	5.64e-05	tgcctctgggcg
MA0810.1	TFAP2A(var.2)	human ci-INS	558	569	6.01e-07	tgccccggggcg
MA0872.1	TFAP2A(var.3)	mouse ci-Ins2	182	194	3.71e-05	tgccttaaggggt
MA0811.1	TFAP2B	human ci-INS	31	42	2.31e-05	cgcccccagcca
MA0811.1	TFAP2B	human ci-INS	381	392	3.83e-05	tgcctctgggcg
MA0811.1	TFAP2B	human ci-INS	558	569	7.46e-07	tgccccggggcg
MA0811.1	TFAP2B	mouse ci-Ins2	182	193	7.31e-05	tgccttaagggg
MA0813.1	TFAP2B(var.3)	mouse ci-Ins2	182	194	3.93e-05	tgccttaaggggt
MA0524.2	TFAP2C	human ci-INS	31	42	2.7e-05	cgcccccagcca
MA0524.2	TFAP2C	human ci-INS	381	392	5.21e-05	tgcctctgggcg
MA0524.2	TFAP2C	human ci-INS	558	569	9.5e-07	tgccccggggcg

MA0524.2	TFAP2C	mouse ci-Ins2	182	193	7.34e-05	tgccttaagggg
MA0815.1	TFAP2C(var.3)	mouse ci-Ins2	182	194	3.11e-05	tgccttaaggggt
MA0691.1	TFAP4	human ci-INS	529	538	7.63e-05	atcacctgtt
MA0796.1	TGIF1	mouse ci-Ins2	153	164	1.16e-05	tggcagctgcca
MA0797.1	TGIF2	mouse ci-Ins2	153	164	5.79e-06	tggcagctgcca
MA0597.1	THAP1	rat ci-Ins2	157	165	9.23e-05	ctgccagga
MA0597.1	THAP1	mouse ci-Ins2	159	167	9.23e-05	ctgccagga
MA0633.1	Twist2	rat ci-Ins2	137	146	4.75e-05	gacatatgta
MA0633.1	Twist2	rat ci-Ins2	299	308	1.43e-05	cccatatgtc
MA0633.1	Twist2	mouse ci-Ins2	139	148	4.75e-05	gacatatgta
MA0633.1	Twist2	mouse ci-Ins2	300	309	6.98e-05	cccatgtgtc
MA0093.2	USF1	mouse ci-Ins2	215	225	2.7e-05	gacatgtgact
MA0093.2	USF1	mouse ci-Ins2	300	310	5.01e-05	cccatgtgtcc
MA0526.1	USF2	mouse ci-Ins2	215	225	9.18e-06	gacatgtgact
MA0693.1	Vdr	human ci-INS	355	370	1.66e-05	ggggtcacagggtgcc
MA0095.2	YY1	rat ci-Ins2	146	157	6.79e-05	aaacatggtagc
MA0095.2	YY1	mouse ci-Ins2	148	159	1.07e-05	aaacatggcagc
MA0146.2	Zfx	human ci-INS	450	463	8.38e-05	tgtcgccaggcctc
MA0146.2	Zfx	human ci-INS	592	605	1.8e-05	ggcgttggggcctg
MA0696.1	ZIC1	human ci-INS	41	54	7.01e-05	cacccctgctcct
MA0696.1	ZIC1	human ci-INS	367	380	4.27e-05	tgccccacgctgcc
MA0696.1	ZIC1	mouse ci-Ins2	350	363	1.88e-05	tgccacctgcaggg
MA0697.1	ZIC3	human ci-INS	38	52	6.84e-05	agccacccctgctc
MA0697.1	ZIC3	mouse ci-Ins2	350	364	5.32e-05	tgccacctgcagggg
MA0751.1	ZIC4	human ci-INS	367	381	4.2e-05	tgccccacgctgcct
MA0751.1	ZIC4	mouse ci-Ins2	350	364	2.44e-05	tgccacctgcagggg
MA0528.1	ZNF263	human ci-INS	72	92	9.07e-06	tgggcagaagggggcaggagg
MA0528.1	ZNF263	human ci-INS	563	583	4.05e-05	cggggcgggggaaggaggtgg
MA0528.1	ZNF263	human ci-INS	564	584	2.26e-05	ggggcgggggaaggaggtggg
MA0528.1	ZNF263	human ci-INS	573	593	3.05e-06	gaaggaggtgggacatgtggg
MA0528.1	ZNF263	rat ci-Ins2	370	390	6.04e-05	aggggataaagtgagagaaag

Supplementary Table 6 | Transcription factors targeting genes differentially expressed after ci-Ins2 or ci-INS inhibition.

The analyzed genes are shown in Fig. 6

The hypergeometric test *p* value was obtained by using the Enrichr webserver.

Term	*p* value	Adjusted *p* value	Z-score	Combined score	Genes
EGR1 (mouse)	0.0052	0.14	-1.64	8.65	RIMS2;UNC13A;SIK1;CACNA1D;ATP1A1
FOXA1 (human)	0.0200	0.28	-1.65	6.45	RIMS2;GIPR;ATP1B3;ATP1A1
FOXF1 (human)	0.0155	0.28	-1.69	7.02	RIMS2;SYT4;GIPR;RAPGEF4
GLI2 (human)	0.0204	0.28	-1.58	6.13	MIDN;SLC12A5;CACNA1D;RAPGEF4
MYB (human)	0.0179	0.28	-1.65	6.62	RIMS2;SYT4;PCLO;RAPGEF4
NME2 (human)	0.0332	0.32	-1.79	6.09	RIMS2
PCBP1 (human)	0.0024	0.10	-1.73	10.44	RIMS2;SIK1;CACNA1D;ATP1A1;SYT7
RBPJ (human)	0.0238	0.29	-1.63	6.08	RIMS2;PCLO;CACNA1D;ATP1A1
SMAD3 (human)	0.0318	0.32	-1.74	6.01	SYT7
SMAD4 (mouse)	0.0262	0.29	-1.50	5.48	RIMS2;ATP1B3;CACNA1D;SYT7
SP1 (human)	0.0003	0.03	-1.75	13.97	RIMS2;MIDN;SIK1;CACNA1D;ATP1A1;SYT7
SP1 (mouse)	0.0248	0.29	-1.47	5.45	RIMS2;MIDN;SIK1;ATP1B3;CACNA1D
SP3 (human)	0.0003	0.03	-1.76	14.57	RIMS2;MIDN;SIK1;CACNA1D;ATP1A1;SYT7
TCFAP2A (human)	0.0025	0.10	-1.73	10.35	RIMS2;MIDN;SIK1;CACNA1D;SYT7
TEAD4 (human)	0.0156	0.28	-1.64	6.81	RIMS2;MIDN;SIK1;CACNA1D
TFAP2A (human)	0.0393	0.34	-1.54	5.00	RIMS2;MIDN;SLC12A5;SIK1;CACNA1D;ATP1A1
WT1 (human)	0.0410	0.34	-1.37	4.39	RIMS2;SIK1;ATP1B3;CACNA1D;SYT7
ZBTB7A (human)	0.0164	0.28	-1.63	6.69	RIMS2;SIK1;CACNA1D;RAPGEF4
ZNF148 (human)	0.0048	0.14	-1.69	8.99	RIMS2;MIDN;GIPR;ATP1B3;CACNA1D
ZNF281 (human)	0.0349	0.32	-1.62	5.45	RIMS2;ATP1B3;CACNA1D

Supplementary Table 7 | Predicted binding sites of RNA-binding proteins in ci-Ins2 and ci-INS sequences.
Lariat ci-Ins2 and ci-INS sequences are shown in the Fig. 1b sheet of the Excel Source Data file.
The sequence match *p* value was calculated with FIMO's dynamic programming algorithm.

motif_id	motif_name	sequence_name	start	end	*p* _value	matched_sequence
M012_0.6	CPEB2	human ci-INS	120	126	9.66E-05	ctttttt
M149_0.6	CPEB4	human ci-INS	120	126	9.66E-05	ctttttt
M141_0.6	ESRP2	human ci-INS	512	518	9.74E-05	tggggag
M016_0.6	FMR1	human ci-INS	502	508	4.39E-05	ggacagg
M296_0.6	IGF2BP1	rat ci-Ins2	339	349	7.91E-05	atgcaaccctt
M153_0.6	LIN28A	human ci-INS	409	415	4.39E-05	cggagga
M144_0.6	PABPC3	mouse ci-Ins2	376	382	6.51E-05	gaaaacc
M109_0.6	RBM14	human ci-INS	565	571	8.88E-05	gggcggg
M145_0.6	RBM5	human ci-INS	573	579	5.35E-05	gaaggag
M145_0.6	RBM5	rat ci-Ins2	164	170	5.35E-05	gaaggag
M347_0.6	SNRPA	human ci-INS	73	83	2.65E-05	gggcagaaggg
M347_0.6	SNRPA	human ci-INS	83	93	4.52E-05	gggcaggaggc
M348_0.6	SNRPA	human ci-INS	481	487	6.51E-05	aggagat
M104_0.6	SRSF1	human ci-INS	410	416	4.39E-05	ggaggag
M019_0.6	SRSF10	rat ci-Ins2	383	389	7.93E-05	agagaaa
M070_0.6	SRSF2	human ci-INS	481	488	5.61E-05	aggagatg
M065_0.6	SRSF9	human ci-INS	572	578	5.35E-05	ggaagga
M065_0.6	SRSF9	rat ci-Ins2	163	169	5.35E-05	ggaagga
M074_0.6	TARDBP	mouse ci-Ins2	165	172	5.04E-05	ggaatgag

Supplementary Table 8 | Genes differentially regulated in TDP-43 and ci-Ins2 knockdown.
Genes differentially regulated in TDP-43 knockdown are available in GEO accession GSE125424 and GSE134699 for ci-Ins2 knockdown.

ENSEMBL ID	Gene name	direction of change	
		KD ci-Ins2	KD TDP-43
ENSRNOG00000004806	Strn	down	down
ENSRNOG00000001516	Rapgef4	down	down
ENSRNOG00000005711	Ptprd	down	down
ENSRNOG00000003723	Wdr26	down	down
ENSRNOG00000014856	Etnk1	down	down
ENSRNOG00000004232	G2e3	down	down
ENSRNOG00000011150	Arsb	down	down
ENSRNOG00000056703	Atrx	down	down
ENSRNOG00000028156	Pld1	down	down
ENSRNOG00000024602	Plekha7	down	down
ENSRNOG00000005726	Pclo	down	down
ENSRNOG00000007682	Gria3	down	down
ENSRNOG00000061262	Huwe1	down	down
ENSRNOG00000018183	Ubr4	down	down
ENSRNOG00000007090	Cacna1c	down	down
ENSRNOG00000027191	Birc6	down	down
ENSRNOG00000021718	Cspp1	down	down
ENSRNOG00000010964	Akap13	down	down
ENSRNOG00000052003	Mkrn2os	up	down
ENSRNOG00000002079	Mapk10	down	up
ENSRNOG00000025612	Sez6l	down	up
ENSRNOG00000007733	Arhgef9	down	up
ENSRNOG00000004932	Sel1l3	down	up
ENSRNOG00000010299	Eps15	down	up
ENSRNOG00000032576	Nlgn1	down	up
ENSRNOG00000019807	Sufu	down	up
ENSRNOG00000006569	Itgb8	down	up
ENSRNOG00000001424	Cux1	down	up
ENSRNOG00000042951	Xrn1	down	up
ENSRNOG00000026432	Syt7	down	up

Supplementary Table 9 | Gene ontology enrichment of genes differentially regulated in both TDP-43 and ci-Ins2 knockdown conditions.
Source Data are available in GEO accession GSE125424 for TDP-43 knockdown and GSE134699 for ci-Ins2 knockdown.

Category	Term	Count	%	*p* value	Genes	List Total	Pop Hits	Pop Total	Fold Enrichment
GOTERM_BP_	GO:0017156~calcium ion regulated exocytos	2	11.7647059	0.01529369	ENSRNOG000	16	18	17535	121.7708333
GOTERM_BP_	GO:0007507~heart development	3	17.6470588	0.02451569	ENSRNOG000	16	288	17535	11.41601562
GOTERM_BP_	GO:0030073~insulin secretion	2	11.7647059	0.02953702	ENSRNOG000	16	35	17535	62.625
GOTERM_BP_	GO:0000209~protein polyubiquitination	2	11.7647059	0.08854173	ENSRNOG000	16	108	17535	20.29513889

Supplementary Table 10 | Human islet donor characteristics.
Human islets from EXODIAB Sweden were handpicked after isolation. Hence, their purity is estimated to be >90%.

Sample	Age (years)	Gender	BMI (Kg/m^2)	HbA1c (%)	Source of islets	Diabetes	Islet purity (%)
1	73	Male	26.2	5.4	EXODIAB Sweden	No	70
2	54	Male	28.4	5.8	EXODIAB Sweden	No	40
3	68	Female	21.5	5.2	EXODIAB Sweden	No	80
4	53	Female	25.7	5.5	EXODIAB Sweden	No	35
5	57	Male	23.7	5.3	EXODIAB Sweden	No	60
6	58	Female	25.7	5.1	EXODIAB Sweden	No	75
7	49	Female	33.4	5.8	EXODIAB Sweden	No	60
8	49	Female	27.8	5.7	EXODIAB Sweden	No	95
9	57	Male	29.9	5.4	EXODIAB Sweden	No	75
10	44	Female	29.4	6.9	EXODIAB Sweden	Yes	70
11	41	Male	22.5	6.8	EXODIAB Sweden	Yes	40
12	58	Male	24.5	5.8	EXODIAB Sweden	Yes	65
13	57	Female	34.6	6.8	EXODIAB Sweden	Yes	75
14	43	Female	28.7	7	EXODIAB Sweden	Yes	75
15	62	Male	34.9	6.4	EXODIAB Sweden	Yes	30
16	65	Male	27.8	7.2	EXODIAB Sweden	Yes	85
17	62	Male	24.8	N/A	EXODIAB Sweden	Yes	90
18	67	Female	22.9	6.3	EXODIAB Sweden	Yes	90
19	69	Female	33.1	N/A	DCEM Italy	No	70
20	60	Male	19.1	N/A	CEED France	No	85
21	82	Female	21.3	N/A	DCEM Italy	No	80
22	46	Male	27.2	N/A	ECIT Switzerland	No	75
23	69	Female	36.1	5.1	CEED France	No	90
24	58	Male	25.9	5.3	CEED France	No	90
25	71	Female	27.3	N/A	DCEM Italy	No	50
26	86	Female	20.5	N/A	CEED France	No	80

Supplementary Table 11 | Primers used for qPCR amplification.

OJ = Over junction primers

Target	Species	Forward primer (5'-3')	Reverse primer (5'-3')
18S	Human	ggaattattccccatgaacg	ggcctcactaaaccatccaa
Atp1a1	Rat	ttggagagcgtgtgctaggt	aatggctttggctgtgattg
ATP1A1	Human	ctacctggcttgctctgtcc	cgacgattttggcgtatctt
Atp1b3	Rat	actttttcatcaggagggtcca	agctccatctgggactaactcg
ATP1B3	Human	cattcacgatgtgggttatgct	ttcaaaaagtgctccatcagga
Cacna1d	Rat	caggtggatcgctcagagtcta	ctgtctgcactcctctgcttgt
CACNA1D	Human	tttgtgtcagcaacaaaacacg	agccaggaccaaagtcctgtag
circHIPK3	Mouse	ggtgggtagaccaagacttgtga	tatgttggtggatcctgttcgg
ciRS-7	Mouse	tgtatcggcgttttgacattca	actggaagactctgaggttgt
Gapdh	Mouse	cagtgagcttcccgttca	atcactgccacccagaagac
Gcg	Rat	gaagttaccgccctgagatt	cgcatttatgacaaagggttc
Gipr	Rat	gtttgcatatgccagaggacag	ctcactgcccctagtccctaga
GIPR	Human	atgcctggctaatttttgcatt	ctgcccctgataatttgtcctc
Hprt1	Mouse/Rat	agtcccagcgtcgtgattag	aatccagcaggtcagcaaag
HPRT1	Human	ccctggcgtcgtgattagtg	gctacaatgtgatggcctccc
INS	Human	accagctggagaactactgcaa	tggttcaagggctttattccat
INS intron 2 (divergent)	Human	ccctgactgtgtcctcctgt	agcctcctgcccccttct
INS intron 2 lariat (ci-INS)	Human	ctgtgtccgtgagccaacc (OJ)	tgcccaccctctgatgtatct
Ins2	Mouse	tttgtcaagcagcacctttgt	gctggtgcagcactgatctac
Ins2	Rat	tggggagcgtggattcttct	cagaggggtggacagggtag
Ins2 intron 1	Rat	tatgtactctccagggtgggc	cacagcgtccctcaaatccc
Ins2 intron 2 (divergent)	Mouse	gtgtccatccatgaccagtgact	cccaagtgtgaagaaaaccaggg
Ins2 intron 2 (divergent)	Rat	tgagagaaagcctgggctaa	cactccttcctggcagctac
Ins2 intron 2 lariat (ci-Ins2)	Mouse	cctatgtgtctgtgagttctgc (OJ)	gtcccaagttactaccaccaag
Ins2 intron 2 lariat (ci-Ins2)	Rat	tgtccattcatgaccagtgac	gagcagagcttacagacacatag (OJ)
Malat1	Mouse	tagagggtgggcttttgttg	actgccaggctggttatgac
Micu1	Rat	tgggactttgctctacccaaat	agagcctagcggaacactgact
MICU1	Human	gcaggcagaaagaagtctgtga	tgaatcttgctgtgttcccact
Midn	Rat	tcttctcagggaccttctctgg	gtagttttgggggtgaggtcag
MIDN	Human	gctttgcttcctacctccttca	gccatccagagagactgctaca
Pclo	Rat	cccgttaagtgctgtctcctct	ggttgctgtaagggaggttttg
PCLO	Human	aagtggttcagaaggagcaagg	gtctgggtaggaccctgaattg
Pld1	Rat	accaaaaggggtcactggagta	catcctcctttggatgtgactg
PLD1	Human	ttagctgctgggttgattgaaa	ccctggtgagaggcgttaatac
PPIA	Human	tctgcccaccttaacagacc	acccagggaatacgtaaccag
Rapgef4	Mouse	acgctgcacactctcagtcct	ccgcataaacaaatctgacgaa
Rapgef4	Rat	gattgcaaatactgccagaacg	gccttcgaggctctaatctgtg
RAPGEF4	Human	gctgcattcagcttaccagcta	acgtgggccttattcaacactt
Rims2	Rat	cctgcttccctagggtggtt	gacagcctggatgattttcctc
RIMS2	Human	cctgcttccctagggtggtt	catgacagccaggatgattttc
Sik1	Rat	tcctgggactgaacaagatcaa	agtcagatgagacggctgagtg
SIK1	Human	gctgctgacgtttatgcatttc	caaaacccaaaaagcctaggtg
Slc12a5	Rat	caactacaccaacctgcctcag	agccgcagaaagaggataacac
SLC12A5	Human	ggcacctttctggaaaatcatc	caatgaagttcctccacgtctg
Syt4	Rat	taccagccatggatgaacaatc	caaaactcaggacggtgaagtg
SYT4	Human	gatattccttgtgagggccttg	tgtctcctggggtagtcacaga
Syt7	Mouse	ccagaagttcgagggagtcct	tgcgtgagtagctcagagcag
Syt7	Rat	ctggtctctgcaatcatcacc	gtattcacagccttccctcct
SYT7	Human	ctggtctctgccatcatcac	ccaaggaattcttgtagcgttt
Unc13a	Mouse/Rat	accttaacccagtgtgggaaga	tgtctgccctaggaagtcatca
UNC13A	Human	ttttggatggctggaaagaaat	gttctagcctgggagtcaggaa
