# Supplementary material for: A circular RNA generated from an intron of the insulin gene controls insulin secretion
Source: Nat Commun. 2020 Nov 5;11:5611. doi: 10.1038/s41467-020-19381-w (PMC7644714; doi:10.1038/s41467-020-19381-w)
Supplement: Supplementary file 2 — Reporting Summary [file 41467_2020_19381_MOESM2_ESM.pdf]

nature research

Corresponding author(s): Dr. Romano Regazzi

Last updated by author(s): Sep 23, 2020

Reporting Summary

Nature Research wishes to improve the reproducibility of the work that we publish. This form provides structure for consistency and transparency in reporting. For further information on Nature Research policies, see Authors & Referees and the Editorial Policy Checklist .

Statistics

For all statistical analyses, confirm that the following items are present in the figure legend, table legend, main text, or Methods section.

n/a	Confirmed	
☐	☒	The exact sample size (*n*) for each experimental group/condition, given as a discrete number and unit of measurement
☐	☒	A statement on whether measurements were taken from distinct samples or whether the same sample was measured repeatedly
☐	☒	The statistical test(s) used AND whether they are one- or two-sided<br>*Only common tests should be described solely by name; describe more complex techniques in the Methods section.*
☐	☒	A description of all covariates tested
☐	☒	A description of any assumptions or corrections, such as tests of normality and adjustment for multiple comparisons
☐	☒	A full description of the statistical parameters including central tendency (e.g. means) or other basic estimates (e.g. regression coefficient) AND variation (e.g. standard deviation) or associated estimates of uncertainty (e.g. confidence intervals)
☐	☒	For null hypothesis testing, the test statistic (e.g. *F*, *t*, *r*) with confidence intervals, effect sizes, degrees of freedom and *P* value noted<br>*Give P values as exact values whenever suitable.*
☒	☐	For Bayesian analysis, information on the choice of priors and Markov chain Monte Carlo settings
☒	☐	For hierarchical and complex designs, identification of the appropriate level for tests and full reporting of outcomes
☐	☒	Estimates of effect sizes (e.g. Cohen's *d*, Pearson's *r*), indicating how they were calculated

Our web collection on statistics for biologists contains articles on many of the points above.

Software and code

Policy information about availability of computer code

Data collection	No software was used.
Data analysis	CIRI2, find_circ, Chromas (2.6.5), Andor iQ (2.5.1), STAR (2.5.3a), RSEM (1.3.0), HISAT (2.1.0), HTseq (0.10.0), DESeq2 (v.1.12.4), DAVID webserver (6.8), FIMO (5.1.0), Enrichr webserver (2016), GraphPad Prism 7, IBM SPSS Statistics 25.

For manuscripts utilizing custom algorithms or software that are central to the research but not yet described in published literature, software must be made available to editors/reviewers. We strongly encourage code deposition in a community repository (e.g. GitHub). See the Nature Research guidelines for submitting code & software for further information.

Data

Policy information about availability of data

All manuscripts must include a data availability statement. This statement should provide the following information, where applicable:

- Accession codes, unique identifiers, or web links for publicly available datasets
- A list of figures that have associated raw data
- A description of any restrictions on data availability

All data supporting the findings of this study are available within the article and its supplementary information files or from the corresponding author upon request. RNA sequencing data of ci-Ins2 knockdown in rat islet cells and annotated circRNAs are available under the Gene Expression Omnibus (GEO) accession code GSE134699 in https://www.ncbi.nlm.nih.gov/geo/query/acc.cgi?acc=GSE134699; high-troughput RNA-sequencing data from mouse islets under GEO accession GSE92602 in https://www.ncbi.nlm.nih.gov/geo/query/acc.cgi?acc=GSE92602 (Ref #10) and microarray data of MIN6 cells depleted from TDP-43 under GEO accession GSE125424 in https://www.ncbi.nlm.nih.gov/geo/query/acc.cgi (Ref #68). ATtRACT database is available in https://attract.cnic.es/ (Ref #72), Jaspar database in http://jaspar.genereg.net/ (Ref #73), ENSEMBL 92, rn6 in http://apr2018.archive.ensembl.org/Rattus_norvegicus/Info/Index, and Rat Transcriptome (rn6), Human Transcriptome (hg38) and Mouse Transcriptome (mm10) in https://genome.ucsc.edu/cgi-bin/hgGateway. The source data underlying Figs. 1a-c, 2a-f, 3a-c, 4a-d, 5a-f, 6b-f, 7a-c, 8a-b, 9a-g are provided as a Source Data file. The source data underlying Supplementary Fig. 2 are provided as a compressed archive file containing the .ab1 raw sequencing files.

Field-specific reporting

Please select the one below that is the best fit for your research. If you are not sure, read the appropriate sections before making your selection.

- [x] Life sciences
- [ ] Behavioural & social sciences
- [ ] Ecological, evolutionary & environmental sciences

For a reference copy of the document with all sections, see nature.com/documents/nr-reporting-summary-flat.pdf

Life sciences study design

All studies must disclose on these points even when the disclosure is negative.

Sample size	No calculation was performed to predetermine sample size. It was based on the quality, functionality, and availability of the samples in order to obtain representative results. The sample sizes were sufficient for the requirements of the respective statistical test.
Data exclusions	No data were excluded.
Replication	Independent experiments including positive and/or negative controls were performed for each analysis. Replication was confirmed.<br>The experiments shown were repeated as follows:<br>1 time: Fig. 1a,c<br>2 times: Fig. 7b,c<br>3 times: Fig. 2c,e (rat ci-Ins2, human ci-INS, human INS), 6e,f, 9a<br>4 times: Fig. 2a,b,d,e (rat Ins2), 3a,b, 8a,b<br>5 times: Fig. 2f, 3c, 4b, 6b(Atp1a1 and Syt7), 9b(db+/+)<br>6 times: Fig. 4a,d, 5a,b,c, 6b(all but Atp1a1 and Syt7),c, 9b(db+/-),c<br>8 times: Fig. 5d,e,f, 6d, 9f(type 2 diabetes),g(type 2 diabetes)<br>9 times: Fig. 4c, 9e,f(control),g(control)
Randomization	Animal studies included control and diabetic animals matched by age and genetic background. Human studies for RNA levels included type 2 diabetes and aged- and BMI-matched non-diabetic control cadaver donors. Human studies for in vitro experiments included non-diabetic cadaver donors of random age, sex, and BMI. Randomization was not required for studies using MIN6 or primary rat islet cells as all experimental groups were obtained from cells of either the same passage or same animal for each biological replicate.
Blinding	Investigators were not blinded to group allocation. Blinding was not relevant since controls were used for the animal and human studies. In addition, most data are supported by data obtained from unbiased methods such as RNA sequencing.

Reporting for specific materials, systems and methods

We require information from authors about some types of materials, experimental systems and methods used in many studies. Here, indicate whether each material, system or method listed is relevant to your study. If you are not sure if a list item applies to your research, read the appropriate section before selecting a response.

Materials & experimental systems

n/a	Involved in the study
☐	☒ Antibodies
☐	☒ Eukaryotic cell lines
☒	☐ Palaeontology
☐	☒ Animals and other organisms
☐	☒ Human research participants
☒	☐ Clinical data

Methods

n/a	Involved in the study
☒	☐ ChIP-seq
☒	☐ Flow cytometry
☒	☐ MRI-based neuroimaging

Antibodies

Antibodies used	Mouse anti-glucagon (Abcam ab10988), guinea pig anti-insulin (Dako A0564), rabbit anti-Ki67 (Abcam ab15580), rabbit anti-TDP-43 (Abcam ab190963), goat anti-mouse IgG Alexa Fluor 555 (Thermo Fisher A-21422), goat anti-guinea pig IgG Alexa Fluor 488 (Thermo Fisher A-11073), goat anti-guinea pig IgG Alexa Fluor 555 (Thermo Fisher A-21435), goat anti-rabbit IgG Alexa Fluor 488 (Thermo Fisher A-11008), goat anti-rabbit HRP-conj. IgG (H+L) (Jackson ImmunoResearch 211-035-109).
Validation	Primary antibodies were obtained from the aforementioned companies. Testing was performed to control for specificity and efficiency. Company websites and literature were consulted for selecting the antibodies according to their use for immunocytochemistry/immunofluorescence (ICC/IF) or western blot (WB). Citations/Validations for ICC/IF referenced by Abcam: Mouse anti-glucagon ab10988: 3, verified by IF and IHC in rat pancreas sections; Rabbit anti-Ki67 ab15580: 73., verified by IHC in mouse spleen, human spleen, human colon, human skin carcinoma, mouse tumor sections and IF in rat retina, HAP1 cells,

mouse trachea, SK-N-SH cells, mouse olfactory bulb, human skin, human umbilical artery endothelial cells, HeLa cells and MEF1 cells, as well as, knockout samples. Citations for ICC referenced by Labome for guinea pig anti-insulin (Dako A0564): 12, with the following publications using this antibody for ICC in rat tissues: Zhang et al, J Cell Bio, 2017; Hayes et al, Mol Cell Biol, 2016; Shen et al, Nat commun, 2015.

Eukaryotic cell lines

Policy information about cell lines

Cell line source(s)	The MIN6B1 cells were kindly provided by Dr. Philippe Halban (Ref# 61).
Authentication	The secretory capacity of MIN6B1 cells to release insulin in response to glucose or other secretagogues was verified after each thaw and frequently during passaging. The cell line was not further authenticated.
Mycoplasma contamination	MIN6B1 cells tested negative for mycoplasma contamination.
Commonly misidentified lines (See ICLAC register)	No commonly misidentified cell lines were used in the study.

Animals and other organisms

Policy information about studies involving animals; ARRIVE guidelines recommended for reporting animal research

Laboratory animals	Male Wistar rats and C57Bl/6N mice aged 12 weeks, male C57BL/KsJ db/db, C57BL/6J ob/ob, and their respective control db +/- and ob -/- mice aged 13-16 weeks, and female Goto-Kakizaki (GK/MolTac) and control Wistar rats aged 8 weeks.
Wild animals	The study did not involve wild animals.
Field-collected samples	The study did not involve samples collected from the field.
Ethics oversight	Procedures were performed in agreement with the NIH guidelines and approved by the Swiss research council and veterinary offices, the national health and medical research council of Australia, and the ethics committee of Lund University.

Note that full information on the approval of the study protocol must also be provided in the manuscript.

Human research participants

Policy information about studies involving human research participants

Population characteristics	Male and female type 2 diabetes and non-diabetic cadaver donors aged 41-82 years.
Recruitment	Islets from cadaver donors were provided by the Human Tissue Lab of EXODIAB / Lund University Diabetes Centre through the Nordic Network for Islet Transplantation of Uppsala University, the Centre Européen d'Etude du Diabète of Strasbourg University (isolation protocol authorization for scientific research #PFS12-0013), the Department of Clinical and Experimental Medicine of Pisa University, and the European Consortium for Islet Transplantation of Hôpitaux Universitaires de Genève through the JDRF award 31-2008-416 (ECIT Islet for Basic Research program). Informed consent was previously provided by all donors. We have no evidence of any biases.
Ethics oversight	Procedures were approved by the ethics committees of the Uppsala, Strasbourg, Pisa, and Gèneve Universities.

Note that full information on the approval of the study protocol must also be provided in the manuscript.
